# Genetic Variation in the Familial Mediterranean Fever Gene (*MEFV*) and Risk for Crohn's Disease and Ulcerative Colitis

**DOI:** 10.1371/journal.pone.0007154

**Published:** 2009-09-28

**Authors:** Alexandra-Chloé Villani, Mathieu Lemire, Edouard Louis, Mark S. Silverberg, Catherine Collette, Geneviève Fortin, Elaine R. Nimmo, Yannick Renaud, Sébastien Brunet, Cécile Libioulle, Jacques Belaiche, Alain Bitton, Daniel Gaudet, Albert Cohen, Diane Langelier, John D. Rioux, Ian D. R. Arnott, Gary E. Wild, Paul Rutgeerts, Jack Satsangi, Séverine Vermeire, Thomas J. Hudson, Denis Franchimont

**Affiliations:** 1 Department of Gastroenterology, McGill University Health Centre, Montreal General Hospital, Montréal, Québec, Canada; 2 McGill University and Génome Québec Innovation Centre, Montréal, Québec, Canada; 3 Ontario Institute for Cancer Research, MaRS Centre, Toronto, Ontario, Canada; 4 Department of Gastroenterology, Centre Hospitalier Universitaire de Liège, Université de Liège, Liège, Belgium; 5 Department of Gastroenterology, Mount Sinai Hospital IBD Centre, University of Toronto, Toronto, Ontario, Canada; 6 Department of Gastroenterology, Molecular Medicine Centre, University of Edinburgh, Western General Hospital, Edinburgh, United Kingdom; 7 Department of Gastroenterology, McGill University Health Centre, Royal Victoria Hospital, Montréal, Québec, Canada; 8 Department of Medicine, Université de Montréal, Centre de médecine génique communautaire de l'Université de Montréal, Hôpital universitaire de Chicoutimi, Chicoutimi, Québec, Canada; 9 Department of Gastroenterology, Jewish General Hospital, Montréal, Québec, Canada; 10 Department of Gastroenterology, Centre Hospitalier Universitaire de Sherbrooke – Hôtel-Dieu, Sherbrooke, Québec Canada; 11 Department of Medicine, Université de Montréal and Montreal Heart Institute, Research Center, Montréal, Québec, Canada; 12 Department of Gastroenterology, University Hospital Gasthuisberg, Leuven, Belgium; 13 Department of Gastroenterology, Erasme Hospital, Université Libre de Bruxelles, Brussels, Belgium; Cairo University, Egypt

## Abstract

**Background and Aims:**

The familial Mediterranean fever (FMF) gene (*MEFV*) encodes pyrin, a major regulator of the inflammasome platform controlling caspase-1 activation and IL-1β processing. Pyrin has been shown to interact with the gene product of *NLRP3*, NALP3/cryopyrin, also an important active member of the inflammasome. The *NLRP3* region was recently reported to be associated with Crohn's disease (CD) susceptibility. We therefore sought to evaluate *MEFV* as an inflammatory bowel disease (IBD) susceptibility gene.

**Methodology and Results:**

*MEFV* colonic mucosal gene expression was significantly increased in experimental colitis mice models (TNBS *p*<0.0003; DSS *p*<0.006), in biopsies from CD (*p*<0.02) and severe ulcerative colitis (UC) patients (*p*<0.008). Comprehensive genetic screening of the *MEFV* region in the Belgian exploratory sample set (440 CD trios, 137 UC trios, 239 CD cases, 96 UC cases, and 107 healthy controls) identified SNPs located in the *MEFV* 5′ haplotype block that were significantly associated with UC (rs224217; *p* = 0.003; *A* allele frequency: 56% cases, 45% controls), while no CD associations were observed. Sequencing and subsequent genotyping of variants located in this associated haplotype block identified three synonymous variants (*D102D*/rs224225, *G138G*/rs224224, *A165A*/rs224223) and one non-synonymous variant (R202Q/rs224222) located in *MEFV* exon 2 that were significantly associated with UC (rs224222: *p* = 0.0005; *A* allele frequency: 32% in cases, 23% in controls). No consistent associations were observed in additional Canadian (256 CD trios, 91 UC trios) and Scottish (495 UC, 370 controls) sample sets. We note that rs224222 showed marginal association (*p* = 0.012; *G* allele frequency: 82% in cases, 70% in controls) in the Canadian sample, but with a different risk allele. None of the *NLRP3* common variants were associated with UC in the Belgian-Canadian UC samples and no significant interactions were observed between *NLRP3* and *MEFV* that could explain the observed flip-flop of the rs224222 risk allele.

**Conclusion:**

The differences in association levels observed between the sample sets may be a consequence of distinct founder effects or of the relative small sample size of the cohorts evaluated in this study. However, the results suggest that common variants in the *MEFV* region do not contribute to CD and UC susceptibility.

## Introduction

Crohn's disease (CD) and ulcerative colitis (UC) are multifactorial and heterogeneous, chronically relapsing inflammatory bowel diseases (IBD) that are thought to result from a dysregulated mucosal immune response to gut lumen bacterial antigens in a genetically susceptible host [1]. A recent meta-analysis of three large CD genome-wide association studies (GWAS) has reported that well-established associations with CD only accounts for approximately 20% of the genetic variance observed in CD, suggesting that additional genetic contributions have yet to be discovered [2]. Indeed, with the exception of variations within the *NOD2* and *IL23R* genes, established susceptibility alleles have been reported to have relatively modest effects [2]. Using a candidate gene approach, *NLRP3* (Chr.1q44; GenBank AF054176; OMIM 606416) was recently identified as a novel CD susceptibility locus [3] that had not been described in previously published GWAS [4]–[9]. *NLRP3* (previously known as *CIAS1*) is part of the CATERPILLER (CARD (caspase recruitment domain), Transcription Enhancer, R (purine)-binding, Pyrin, Lots of Leucine Repeats) [10] gene family and mutations in some of these genes have been shown to result in severe auto-inflammatory diseases (AIDs) [11]. AIDs represent a spectrum of diseases characterized by recurrent episodes of seemingly unprovoked inflammation that, unlike autoimmune disorders, lack the production of high-titer auto-antibodies or antigen-specific T cells [10]. Gain-of-function mutations in *NLRP3* are associated with three hereditary periodic fever syndromes: Muckle-Wells syndrome, familial cold autoinflammatory syndrome, and neonatal-onset multisystem inflammatory disease [10]–[13]. *NLRP3* encodes NALP3 (also known as cryopyrin). This protein plays a key role in controlling the inflammasome, which is a critical molecular platform regulating caspase-1 activation and interleukin (IL)-1β processing, two key mediators of inflammation [12]–[15].

Recently, the SPRY (also known as B30.2) domain of the protein pyrin, which is encoded by the *MEFV* gene (Chr.16p13.3; GenBank NM_000243; OMIM 608107), has been reported to interact with and modulate the activity of several inflammasome components, including NALP3/cryopyrin, caspase-1, and, its substrate, pro-IL-1β [16]–[18]. Interestingly, *MEFV* missense mutations are implicated in the familial Mediterranean fever (FMF), which is another AID [19]–[22].

Additional clinical and epidemiological evidence supported *MEFV* as a potential IBD candidate gene. IBD and FMF share common clinical and biologic features; they are both inflammatory disorders characterized by the same chronic relapsing behavior, infiltration by neutrophils at the site of injury, and abnormal regulation of apoptosis [23]–[24]. Moreover, FMF affects mainly ethnic groups surrounding the Mediterranean Sea (e.g. non-Ashkenazi Jews, Armenians, Turks, and Arabs) and two small cohort studies from that region have reported a higher prevalence of IBD, with particularly severe symptoms in FMF non-Ashkenazi Jewish patients, suggesting possible common underlying mechanisms of inflammation [25]–[26]. Additional studies have suggested that *MEFV* rare missense causative mutations have a potential modifying effect in IBD patients [27]–[30].

This clinical and epidemiological evidence linking IBD to the *MEFV* gene, together with the co-localization of the *MEFV* and *NLRP3* gene products (pyrin and NALP3, respectively) within the same signaling pathway, suggested that *MEFV* could also contribute to CD and/or UC susceptibility. We therefore explored whether *MEFV* expression was regulated in IBD experimental models of colitis and whether *MEFV* variants were associated with CD and/or UC susceptibility.

## Results

### 1. MEFV expression is increased in IBD experimental models of colitis and in colonic mucosa from IBD patients

**Figure 1:**
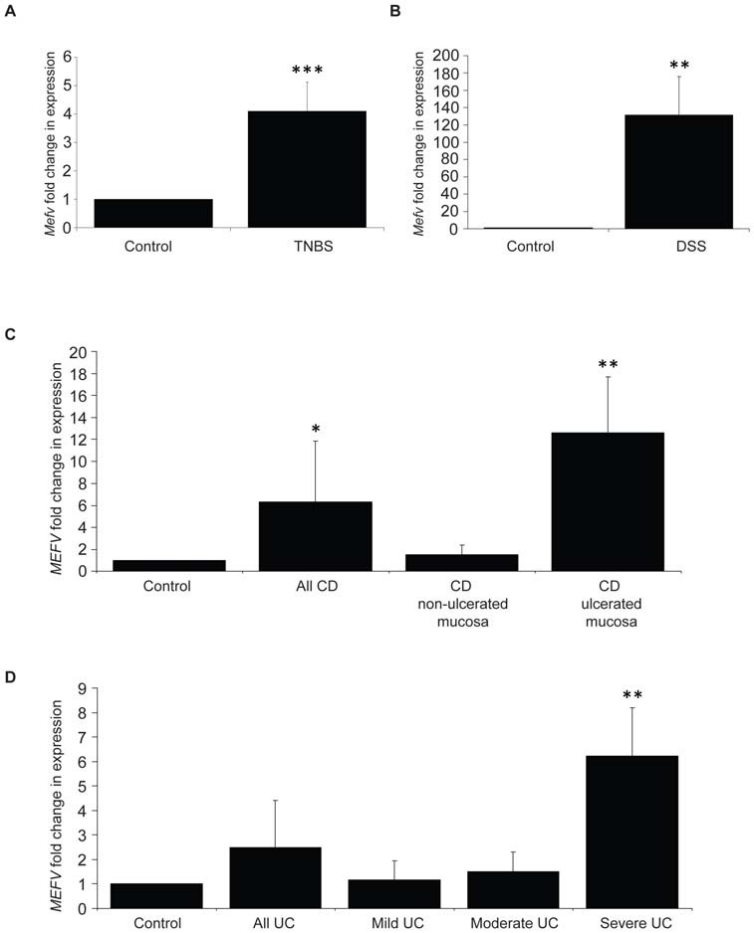
Level of MEFV mRNA expression in inflamed colitis tissues compared to healthy controls. (a, b) *MEFV* expression was assessed by quantitative real-time PCR in (a, b) healthy mice colons (n = 7), in colons from (a) TNBS-induced (n = 8) and (b) DSS-induced (n = 4) colitis mice models, as well as in (c, d) healthy human (n = 25), (c) CD (n = 16) and (d) UC (n = 17) colonic specimens. *MEFV* expression appeared to correlate with disease severity in both CD (c: 9 CD with mild inflammation and 7 CD with severe inflammation) and UC (d: 6 UC with mild inflammation, 7 UC with moderate inflammation and 4UC with severe inflammation) samples. (a–d) Expression was normalized to *18S* gene expression and each bar represents the mean value±S.D. (*) = *p*<0.05 compared to healthy colonic specimens, considered as the arbitrary baseline = 1.

To further support our candidate gene selection, we first evaluated *MEFV* expression in different models of experimental colitis. *Mefv* expression is significantly increased in trinitrobenzene sulfonic acid (TNBS)-induced (fold change  = 4.10±1.02; *p*<0.0003) (Figure 1A) and in dextran sulfate sodium (DSS)-induced (fold change = 131.74±44.14; *p*<0.006) (Figure 1B) colitis mice models as compared to colonic tissues from control mice (arbitrary baseline = 1). In human tissue biopsies, *MEFV* expression is significantly increased in ulcerated colonic mucosa from CD patients compared to healthy controls (fold change = 6.35±5.45; *p*<0.02) (Figure 1C), but not from UC patients (fold change = 2.50±1.90; *p*<0.27) (Figure 1D). However, stratification by severity of disease, as defined by the presence of endoscopic lesions (CD) and Mayo sub-endoscopic criteria (UC), shows that *MEFV* expression is upregulated in both severely affected CD (fold change = 12.58±5.11; *p*<0.001) (Figure 1C) and UC (fold change = 6.23±1.97; *p*<0.008) (Figure 1D) patients compared to healthy controls.

### 2. MEFV mutations in exon 10 do not contribute to CD and UC susceptibility

FMF is thought to be secondary to missense mutations in *MEFV*. Although these mutations are found throughout the gene, five sequence alterations in *MEFV* represent the majority of FMF chromosomes, four of which are clustered in exon 10 (i.e. *M680I*/rs28940580, *M694V*/rs61752717, *M694I*/rs28940578, and *V726A*/rs28940579) [11], [20]–[22]. This exon was sequenced in 47 CD patients, 47 UC patients and 94 controls. Only three individuals (2 CD and 1 UC patients) carrying three different mutations (*R652H*/rs28940581, *M694V*/rs61752717, and *V726A*/rs28940579 respectively) were observed. None of the exon 10 variants could link IBD with *MEFV*. The low frequency of these observed mutations confirms previous reports studying the prevalence and association of these mutations in IBD patients [25]–[30].

### 3. Common SNPs located in MEFV 5′ region are significantly associated with IBD in the Belgian exploratory sample set

We subsequently sought to evaluate *MEFV* common variants for their association with IBD. We first investigated a 98 kb region spanning chromosome 16p13.3 (3220975–3318978) (NCBI Build 35, hg17), including *MEFV* (14.6 kb). A pairwise tagging approach [31] was used to select single nucleotide polymorphisms (SNPs), using data from HapMap Public Release #22 (*r^2^*≥0.8) (minor allele frequency (MAF) ≥0.05). The SNP panel was enriched with SNPs located in functional domains, as well as with SNPs selected from dbSNP Build126 in regions with lower coverage. A total of 30 informative SNPs in the *MEFV* region were genotyped in 440 Belgian CD trios and 137 UC trios (Table 1).

**Table 1:** Subjects examined as part of the MEFV candidate gene study.

	Number of subjects			
	IBD	CD	UC	Control
**Exploratory Combined Belgian Cohort**				
1- Leuven Trios	389	286	103	N/A
2a- Liege Trios	188	154	34	N/A
2b- Liege C/C^1^	335	239	96	107
Total Exploratory Cohort	912	679	233	107
**Replication Cohorts:**				
**Canadian Combined Cohort**				
3- Québec Trios^2^	170	133	37	N/A
4- Toronto Trios^3^	177	123	54	N/A
Total Canadian Cohort	347	256	91	N/A
**Scottish Cohort**				
5- Edinburgh (Scottish) C/C^1,4^	495	0	495	370
Total Replication Cohort	842	256	586	370

1 C/C refers to case control sample set.

2 Includes 27 CD trios and 13 UC trios of Jewish ancestry.

3 Includes 24 CD trios and 10 UC trios of Jewish ancestry; includes also 15 non-European probands, which were excluded from the analysis.

4 Includes 1 UC case of Jewish ancestry; includes also 14 cases and 11 controls of non-European descent, which were excluded from the analysis.

**Figure 2:**
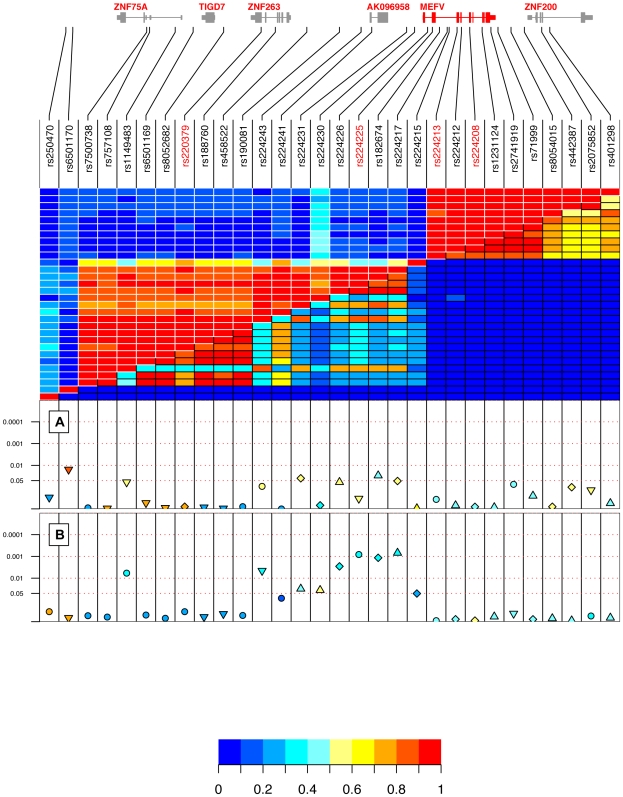
Exploratory phase association results of the SNP panel screened in the combined Belgian CD and UC trios sample sets. Shown above are the SNPs with their positions in the genes and the LD structure between them. SNPs in red are exonic. The upper left portion of the coloured matrix is *D'* and the lower right portion is *r^2^*. Data for the first SNP is represented on the left column and the bottom row of the matrix. In the lower panel are reported the results from association analysis of the Belgian CD (a), and the Belgian UC (b) samples. Level of significance can be found on the scale located at the bottom left of the figure. *P* value of individual alleles are reported, where the symbols represent the associated allele (▾ = T, • = C, ▴ = A, ♦ = G) and the color scheme represents the value of the LD measures and the allele frequency.

As previously reported [32], we observed two regions of high linkage disequilirium (LD) separated by a recombination “hotspot” within *MEFV* intron 2 (Figure 2). The 5′ haplotype block includes the promoter to *MEFV* intron 2 region, three known genes (*ZNF263*, *TIGD7*, *ZNF75A*), and one hypothetical gene (*AK096958*). The 3′ haplotype block spans *MEFV* intron 2 to the 3′ UTR region of *MEFV*, as well as the region encompassing the promoter to intron 4 of *ZNF200* (Figure 2).

The analysis of Belgian CD trios revealed only nominally significant associations with the *major* alleles (i.e. allele frequency in controls >50%) of four SNPs, three of which are located in the haplotype block encompassing the region covering the *MEFV* promoter to intron 2 and its 5′ flanking region (rs182674(*A*): *p* = 0.029; rs224217(*G*): *p* = 0.052; rs224231(*G*); *p* = 0.039; rs6501170(*T*): *p* = 0.016; the risk nucleotides are indicated in brackets) (Figure 2A, Table S1).

The analysis of Belgian UC trios revealed significant associations (lowest *p* = 0.0007 for rs224217) with eight SNPs (i.e. rs1149483(*C*), rs224243(*T*), rs224231(*A*), rs224230(*A*), rs224226(*G*), rs224225(*C*), rs182674(*G*), and rs224217(*A*)) located in that same 5′ haplotype block also associated with CD (Figure 2B, Table S1). We note that the risk alleles differ for those SNPs associated with both CD and UC. Contrary to the CD associations, the UC associations remained significant after allowing for the total number of tests performed (*p_corrected_* = 0.018 based on 1000 random permutations in 2 diseases; see Methods). These associations with UC were also significant across the two Belgian Centers (rs224217: *p* = 0.038 [Center 1: Leuven]; *p* = 0.0008 [Center 2: Liege]) described in Table 1.

### 4. Sequencing of coding and promoter regions of genes located in the associated 5′ haplotype block

Following the identification of the association signals with CD and UC in the 5′ haplotype block, we sequenced the exons and promoter regions (i.e. 2 kb upstream) of all genes located within this region to exclude the involvement of other genes (i.e. *AK096958*, *ZNF263*, *TIGD7*, *ZNF75A*) aside from *MEFV*. A total of 10 UC patients, 10 CD patients and 10 healthy controls were sequenced, and 9 synonymous and 9 non-synonymous variants were identified. A detailed description of all observed coding variants can be found in Table 2.

**Table 2:** Coding variants uncovered in the MEFV 5′ haplotype block.

Base Change^1^	Amino Acid Change	dbSNP#^2^	Gene	Location
c.77G>A	R26Q	*Novel*	*TIGD7*	exon1
c.1600G>A	V534I	rs34236132	*ZNF263*	exon 6
c.929G>C	C310S	rs220379	*ZNF263*	exon 6
c.742A>C	N248H	*Novel*	*AK096958*	exon1
c.306T>C	D102D	rs224225	*MEFV*	exon 2
c.414A>G	G138G	rs224224	*MEFV*	exon 2
c.442G>C	E148Q	rs3743930	*MEFV*	exon 2
c.495C>A	A165A	rs224223	*MEFV*	exon 2
c.605G>A	R202Q	rs224222	*MEFV*	exon 2
c.942C>T	R314R	rs224213	*MEFV*	exon 3
c.1105C>T	P369S	rs11466023	*MEFV*	exon 3
c.1223G>A	R408Q	rs11466024	*MEFV*	exon 3
c.1422G>A	E474E	rs224208	*MEFV*	exon 5
c.1428A>G	Q476Q	rs224207	*MEFV*	exon 5
c.1530T>C	D510D	rs224206	*MEFV*	exon 5
c.1648C>G	P550A	*Novel*	*MEFV*	exon 7
c.1764G>A	P588P	rs1231122	*MEFV*	exon 9
c.2118G>A	P706P	rs2234939	*MEFV*	exon 10

1 According to the cDNA coding sequence, with +1 from the A of the initiating ATG. Reference sequences are NM_033208 (*TIGD7*), NM_005741 (*ZNF263*), NC_000016 (*AK096958*), and NM_000243 (*MEFV*).

2 dbSNP: www.ncbi.nlm.nih.gov/SNP

### 5. Augmenting the sample size: MEFV common SNPs do not contribute to CD and UC susceptibility

#### 5.1 The Belgian samples

To further validate the Belgian association results, we first augmented the sample size by genotyping additional unrelated Belgian cases and controls (239 CD, 96 UC and 107 shared controls), and combining them with the 440 CD and 137 UC trios previously genotyped. The cases and controls were genotyped for 12 of the 30 exploratory set of SNPs, which were chosen using a pairwise tagging approach [31] to remove the redundancy among the genotyped markers, given the high level of LD between them. Additionally, all Belgian case-control and trio samples were genotyped for the non-synonymous variants observed in the sequencing experiment, which are described in Table 2.

Increasing the sample size in the combined Belgian CD case-control-trio analyses did not reveal more significant associations between the 12 tagging SNPs and CD (Table 3). Moreover, no significant associations were observed between all non-synonymous coding SNPs described in Table 2 and the combined CD Belgian sample set (Table 3).

**Table 3:** Association results of tagging SNPs and MEFV exon 2 coding SNPs in CD sample sets.

		Belgian CD^1^				Canadian CD^2^				Combined CD^3^			
SNP	Position dbSNP130	Allele^4^	Frequency Cases	Frequency Controls	*P* value	Allele^4^	Frequency Cases	Frequency Controls	*P* value	Allele^4^	Frequency Cases	Frequency Controls	*P* value
rs250470	3318978	T	0.27	0.26	0.5166	C	0.72	0.72	1.0000	T	0.27	0.26	0.8085
rs6501170	3317583	T	0.86	0.83	0.1133	T	0.87	0.82	0.0533	T	0.86	0.83	***0.0345***
rs220379/C310S	3279436	G	0.77	0.76	0.6680	G	0.76	0.71	0.1839	G	0.76	0.74	0.4105
rs190081	3263570	T	0.76	0.76	0.9732	T	0.75	0.71	0.1718	T	0.76	0.74	0.6941
rs224243	3259194	C	0.55	0.53	0.5570	T	0.42	0.42	0.8846	C	0.56	0.55	0.9843
rs224241	3257037	T	0.77	0.75	0.2282	T	0.76	0.73	0.3169	T	0.77	0.74	0.4385
rs224230	3248358	A	0.66	0.66	0.7677	G	0.40	0.33	***0.0439***	G	0.36	0.34	0.4493
rs224225/D102D	3244763	T	0.52	0.51	0.8556	T	0.57	0.55	0.6298	T	0.53	0.52	0.7611
rs224224/G138G	3244655	A	0.51	0.51	0.7661	A	0.57	0.55	0.6298	A	0.53	0.53	0.6568
rs224223/A165A	3244574	C	0.52	0.51	0.7818	C	0.57	0.55	0.6298	C	0.53	0.53	0.6832
rs224222/R202Q	3244464	A	0.27	0.26	0.4749	G	0.75	0.70	0.0885	G	0.74	0.72	0.8518
rs224217	3241758	G	0.53	0.51	0.8638	G	0.57	0.56	0.8290	G	0.54	0.53	0.7177
rs224215	3241361	G	0.39	0.39	0.7628	G	0.38	0.37	0.6582	G	0.39	0.38	0.5770
rs1231124	3234679	A	0.48	0.44	0.0822	A	0.49	0.42	0.0563	A	0.48	0.43	0.0549
rs442387	3226119	G	0.54	0.52	0.2781	G	0.56	0.51	0.1526	G	0.55	0.51	0.1015
rs401298	3220975	A	0.40	0.40	0.8400	A	0.41	0.37	0.2182	A	0.40	0.39	0.4608

1 Includes 440 CD trios, 239 CD cases, and 107 healthy controls.

2 Includes 256 CD trios.

3 Includes 696 CD trios, 239 CD cases, and 107 healthy controls.

4 Alleles shown are the alleles seen more frequently in the cases than in the controls.

In the combined Belgian UC case-control-trio analyses, the association significance levels observed with the 12 tagging SNPs were similar to the ones observed in the analysis focussing solely on trios (Table 4). The UC association analysis of the non-synonymous variants from Table 2 revealed no significant associations with *C310S* (previously genotyped rs220379), as well as with the rare coding variants *R26Q* (3 carriers), *V534I*/rs34236132 (11 carriers), and *N248H* (8 carriers). Similarly, no significant associations with coding variants located in the 3′ end region of *MEFV* were detected. However, significant associations were observed with three synonymous variants located in *MEFV* exon 2 (*D102D*/rs224225(*C*), *G138G*/rs224224(*G*), *A165A*/rs224223(*A*)) and UC in the Belgian combined sample set (*p* = 0.0015) (Table 4); these three variants are in perfect LD with each other (*r^2^* = 1.00) and in high LD with tagging SNP rs224217 (*r^2^* = 0.85) (Figure S1). Additionally, the non-synonymous SNP *R202Q*/rs224222 was also significantly associated with UC (*p* = 0.0005; *A* allele frequency: 32% cases, 23% controls) in the Belgian samples. The variant rs224222 is in low LD with the three synonymous variants of exon 2 (*r^2^* = 0.38) and with the tagging SNP rs224217 (*r^2^* = 0.33) (Figure S1).

**Table 4:** Association results of tagging SNPs and MEFV exon 2 coding SNPs in the UC sample sets.

		Belgian UC^1^				Canadian UC^2^				Scottish UC^3^				Combined UC^4^			
SNP	Position dbSNP130	Allele^5^	Frequency Cases	Frequency Controls	*P* value	Allele^5^	Frequency Cases	Frequency Controls	*P* value	Allele^5^	Frequency Cases	Frequency Controls	*P* value	Allele^5^	Frequency Cases	Frequency Controls	*P* value
rs250470	3318978	C	0.75	0.73	0.8473	T	0.26	0.24	0.6681	T	0.21	0.21	0.8969	C	0.77	0.77	0.9156
rs6501170	3317583	C	0.17	0.16	0.7423	T	0.87	0.82	0.3022	C	0.16	0.16	0.9747	C	0.16	0.16	0.8921
rs220379/C310S	3279436	C	0.26	0.23	0.1333	G	0.81	0.70	***0.0457***	C	0.25	0.24	0.8262	G	0.75	0.75	0.8423
rs190081	3263570	C	0.27	0.24	0.1101	T	0.82	0.74	0.0881	C	0.24	0.24	0.8593	C	0.24	0.24	0.8167
rs224243	3259194	T	0.53	0.43	***0.005196***	C	0.57	0.54	0.7098	T	0.48	0.48	0.9977	T	0.49	0.46	0.1282
rs224241	3257037	C	0.26	0.23	***0.0490***	T	0.81	0.71	0.0727	T	0.77	0.77	0.9255	T	0.77	0.76	0.7763
rs224230	3248358	A	0.71	0.66	0.1979	G	0.36	0.33	0.5148	G	0.36	0.35	0.7233	A	0.66	0.66	0.6157
rs224225/D102D	3244763	C	0.56	0.45	***0.001463***	T	0.59	0.54	0.3385	C	0.46	0.45	0.7062	C	0.48	0.45	0.0642
rs224224/G138G	3244655	G	0.55	0.45	***0.002625***	A	0.59	0.54	0.3385	G	0.46	0.45	0.7062	G	0.48	0.45	0.0736
rs224223/A165A	3244574	A	0.55	0.45	***0.001981***	C	0.59	0.54	0.3385	A	0.46	0.44	0.6245	A	0.48	0.45	0.0559
rs224222/R202Q	3244464	A	0.32	0.23	***0.000532***	G	0.82	0.70	***0.0117***	G	0.74	0.74	0.9823	A	0.27	0.26	0.3106
rs224217	3241758	A	0.56	0.45	***0.002545***	G	0.57	0.55	0.7179	G	0.51	0.50	0.8781	A	0.51	0.48	0.1005
rs224215	3241361	G	0.38	0.37	0.4363	A	0.68	0.67	0.7855	A	0.63	0.58	***0.0406***	A	0.63	0.61	0.1738
rs1231124	3234679	A	0.50	0.50	0.5949	A	0.49	0.44	0.3824	A	0.45	0.45	0.9495	A	0.47	0.46	0.5569
rs442387	3226119	G	0.59	0.53	0.0595	G	0.56	0.54	0.7255	G	0.50	0.50	0.9109	G	0.53	0.52	0.2357
rs401298	3220975	A	0.46	0.42	0.2741	A	0.43	0.36	0.3107	G	0.61	0.59	0.3107	G	0.59	0.59	0.8826

1 Includes 137 UC trios, 96 UC cases, and 107 healthy controls.

2 Includes 91 UC trios.

3 Includes 495 UC cases and 370 healthy controls.

4 Includes 228 UC trios, 591 UC cases, and 477 healthy controls.

5 Alleles shown are the alleles seen more frequently in the cases than in the controls.

To assess the reproducibility of these results, we subsequently evaluated the association of the same 12 tagging SNPs and the 4 coding SNPs located in *MEFV* exon 2 region (i.e. rs224225, rs224224, rs224223, and rs224222) in additional samples from Canada (256 CD trios, 91 UC trios) and Scotland (495 UC, 370 controls) (Table 1).

#### 5.2 The Canadian samples

In the Canadian CD sample set, only the minor allele of tagging SNP rs224230, located in the 5′ flanking region of *MEFV*, was significantly associated with CD (*p* = 0.044; *G* allele frequency: 40% cases, 33% controls) (Table 3). None of the 16 SNPs were consistently replicated across the Belgian and Canadian CD sample sets, or in the combined analysis of the Belgian-Canadian CD samples (Table 3), supporting the exclusion of the *MEFV* region as a risk factor contributing to CD susceptibility.

In the Canadian UC sample set, analysis of individual tagging SNPs uncovered one nominally significant association in the *MEFV* 5′ region (rs220379: *p* = 0.046; *G* allele frequency: 81% cases, 70% controls) and one association with coding SNP rs224222 (*p* = 0.012; *G* allele frequency: 82% cases, 70% controls) in exon 2 (Table 4). Contrary to the association in the Belgian samples where the *A* allele of rs224222 was associated with UC susceptibility, the *G* allele is associated with UC in the Canadian samples, and no associations were observed with the three synonymous variants of exon 2 (Table 4). None of these results replicated the initial significant findings observed in the Belgian exploratory UC sample set (Table 4, Figure 2B, Table S1).

#### 5.3 The Scottish samples

In the third UC case-control cohort from Scotland (Edinburgh) (Table 1), only the *A* allele of tagging SNP rs224215 located in *MEFV* intron 2 was significantly associated with UC (*p* = 0.041; *A* allele frequency: 63% cases, 58% controls). None of the associations observed in the Belgian and Canadian UC analyses were replicated in the Scottish sample set (Table 4). No SNPs were significantly associated in the combined analysis of all Belgian-Canadian-Scottish UC samples (Table 4).

### 6. Rare variants in MEFV exon 2

Interestingly, sequencing of exon 2 in all cohorts, primarily as a means to genotype the four exon 2 coding variants (i.e. rs224225, rs224224, rs224223, and rs224222), revealed 15 new rare non-synonymous and 8 synonymous variants (Table S2). Four of them are located on predicted CpG dinucleotides, demonstrating the high potential variability of the *MEFV* exon 2 region. Although these rare variants alone cannot explain the rs224222 associated region, it is interesting to note that of the 8 UC parents carrying a rare mutation, 7 out of 8 transmitted the mutation to affected probands (binomial exact test *p* = 0.07); 3 of these 8 UC parents transmitted the rs224222 risk allele “*A*” to UC probands (*p* = 1.000). Furthermore, 5 UC sporadic cases compared to 3 unaffected individuals carry a rare mutation, and 4 out of the 7 CD parents carrying a rare mutation transmitted the rare allele to affected probands (*p* = 1.000).

### 7. No epistatic interactions are observed between NLRP3 and MEFV in the CD and UC combined Belgian-Canadian sample set

Based on the identification of six common SNPs in the *NLRP3* region contributing to CD susceptibility [3] and reports that the *NLRP3* and *MEFV* gene products interact with each other and are involved in similar pathways, we sought to evaluate whether possible gene-gene interaction between the *MEFV* tagging SNPs and the *NLRP3* six common SNPs could have masked an *MEFV* contribution to CD susceptibility. Using the Belgian-Canadian 639 CD trios, 239 CD cases, and 107 healthy controls commonly genotyped in this study and the *NLRP3* study [3], stratified analyses of *MEFV* variants conditional on each individual's genotypes at *NLRP3* variants did not provide evidence of associations under the possibility of epistasis (Figure S2).

In addition, we also evaluated the association of these six *NLRP3* SNPs in the Belgian and Canadian UC sample sets and subsequently assessed whether gene-gene interactions in the combined Belgian-Canadian UC samples could explain the flip-flop phenomenon observed with rs224222, where different alleles were associated with UC in the Belgian and Canadian samples.

No significant associations were observed between the six *NLRP3* SNPs and UC in the Belgian, Canadian, and combined Belgian-Canadian sample sets when using the commonly genotyped 228 UC trios, 96 UC, and 107 healthy controls (Table S3), nor were any significant interactions found between rs224222 and SNPs in *NLRP3* (Figure S3). Hence, multi-locus effects between the two genes do not explain the observed flip-flop. Additionally, the levels of association for the *MEFV* SNPs conditional on each individual's genotypes at *NLPR3* are in the same order of magnitude than the unconditional levels of association (Figure S3), indicating that epistasis between the two genes is not a mechanism that would be a regulator of the risk of *MEFV* on UC.

## Discussion

Genes, such as *MEFV*, in which mutations lead to severe systemic inflammatory diseases, like the AIDs, play a critical role in the control of inflammation and may represent potential candidates for the onset of chronic inflammatory disorders like CD and UC. To further support our candidate gene selection, we integrated results from mice and human colonic expression studies of *MEFV*. We found *Mefv* expression to be significantly increased in both TNBS-induced colitis mice model, which mimics CD-like intestinal inflammation, and in DSS-induced colitis mice model, which mimics UC-like intestinal inflammation. Gene expression was also increased in inflamed colonic tissues from both CD and UC patients, correlating with severity of inflammation. Fine mapping of the *MEFV* region revealed two haplotype-blocks with a previously reported recombination hotspot located in *MEFV* intron 2 [32]. In our exploratory phase, we report associations of variants located in the *MEFV* 5′ region with both CD and UC in Belgian samples, yet we failed to subsequently validate these associations in the Canadian and Scottish additional sample sets.

The up-regulation of *MEFV* gene expression in the CD and UC patients, as well as in the mouse models, can be explained by the broad involvement of pyrin, the *MEFV* encoded protein, in the regulation of the inflammasome molecular platform and the inflammatory process [12]–[18]. Previously published studies [25]–[28] have excluded the involvement of the *MEFV* gene in CD pathogenesis by looking at specific rare FMF missense causative mutations clustered in exons 2 and 10 in a relatively small number of CD cases. Unlike these previous reports, we carried out a thorough genetic screening of the *MEFV* region in two large CD sample sets and observed no association between common variants in the *MEFV* region and CD. Also, given that common variants in the *NLRP3* region have previously been associated with CD [3] and that the gene products of *NLRP3* and *MEFV* (i.e. NALP3 and pyrin, respectively) are known to interact together in the inflammasome molecular platform [16]–[18], we also perform gene-gene interaction analysis between common variants in the *MEFV* and *NLRP3* region to assess whether such interactions could have masked the *MEFV* contribution to CD pathogenesis. Since no interactions were observed when looking at the large Belgian-Canadian CD sample set and no common variants were consistently associated with CD in the *MEFV* region, we conclude that common variants in the *MEFV* region are unlikely to contribute to CD susceptibility, which is in agreement with previous studies looking at rare FMF causative mutations [25]–[28].

Upon the observation of significant associations between SNPs located in the 5′ block region of *MEFV* and UC in the exploratory phase, we excluded possible coding risk variants and non-*MEFV* genes located in this 5′ block by sequencing all the exonic and promoter regions of the five genes in the region and genotyping the uncovered non-synonymous variants. Only coding SNPs within *MEFV* exon 2 (i.e. rs224225, rs224224, rs224223, and rs224222) were significantly associated with UC in the Belgian samples. However, none of the synonymous variants were associated with UC in the Canadian samples, and we observed opposite rs224222 allele association with UC in the Belgian and Canadian cohorts, suggesting that rs224222 was unlikely to be a causative variant contributing to UC susceptibility.

Such flip-flop associations observed with rs224222 in samples of similar ethnical origins are often regarded as spurious findings, leading to a number of different possible explanations for such observations, like a difference in genetic background and environment [33]–[34]. For example, when attempting to replicate the association of a non-causal allele in LD with the causative variant in two different populations, as it is the case with our Belgian and Canadian sample sets, a difference in LD patterns between the populations could result in inconsistent associations observed across sample sets [33]–[34]. This is not our case, as the Belgian and Canadian UC sample sets displayed very similar LD patterns in the *MEFV* region (data not shown). This inverse association could also indicate the presence of interactions with another risk locus [33]. Complex traits usually result from the interplay of several genetic risk loci and environmental factors. Lin *et al.* have shown that performing single marker analysis without considering the possibility of other genetic risk loci or environmental risk factors correlating with the candidate locus, or the possibility of a multi-locus effect, could also lead to flip-flop associations [33]. To assess this latter possibility, since we knew that the *MEFV* and the *NLRP3* gene product interacted with each other, we genotyped the six SNPs in the *NLRP3* region that had been previously associated with CD [3] in both the Belgian and Canadian UC sample sets and performed gene-gene interaction analysis using these six *NLRP3* SNPs and the 16 SNPs genotyped in the *MEFV* region (i.e. 12 tagging SNPs and 4 coding SNPs in *MEFV* exon 2 region). As no significant interactions were observed between *NLRP3* variants and rs224222, the basis behind the inverse allele associations observed with rs224222 remains unresolved.

Although we have not mapped a causative variant in *MEFV* exon 2, we observed that this exon is enriched with rare mutations that tend to be transmitted to patients, indicating not only the allelic heterogeneity of that region but also its biological importance. However, the distribution of the rs224222 risk allele was not significantly skewed in carriers of these rare mutations.

Of interest, several other groups have reported the contribution of *MEFV* and SNPs in the same 5′ region to the susceptibility of UC and other chronic inflammatory disorder. For example, the alleles of the 4 coding variants of *MEFV* exon 2 (rs224225, rs224224, rs224223 and rs224222: *CGAA*/*G*) that are significantly associated with UC in this study have also been associated with FMF causative rare mutations in Mediterranean populations [32]. A study conducted in a Greek population, focusing precisely on *MEFV* exons 2 and 10, reported an association between UC and a *MEFV* exon 2 haplotype [29]. Yet, the FMF causative mutations were reported to be associated with the other alleles of the exon 2 coding variants (i.e. rs224225, rs224224, rs224223, rs224222: *TACG*) in this population. This latter difference in association could be a consequence of a distinct founder effect or of the relatively small sample size evaluated in their study, which comprised only 25 UC patients, 28 patients with rheumatoid arthritis, and 65 healthy individuals [29]. In agreement with our preliminary UC association results, another study reported the association of six SNPs in the *MEFV* region with juvenile idiopathic arthritis [35]. Among these SNPs, three of them (i.e. rs224217, rs224225, rs224223) located in the *MEFV* 5′ region were also associated in our study. Although of modest size, these studies point towards the same *MEFV* region as being involved in inflammatory processes.

Despite the above-mentioned studies, our UC exploratory association results need to be interpreted with caution. Indeed, the associations were observed in two relatively small UC sample sets (totaling 228 Belgian-Canadian trios, 96 UC cases and 107 healthy controls), and no associations were replicated in our largest UC sample set from Scotland (495 UC cases and 370 controls). Additionally, in the combined analyses of all sample sets, we observed no SNPs significantly associated with UC. Overall, given the lack of consistent replication across all our screened sample sets and the lack of SNP associations in the largest sample set (i.e. combined analysis of all samples), we conclude that *MEFV* is unlikely to contribute to UC susceptibility.

In addition to the size of the sample sets, another possible explanation for the lack of replication may originate from a difference in demographic and clinical characteristics of the UC samples studied. Although no specific genotype-phenotype associations were detected (data not shown), it is worth mentioning that while the mean age of onset of the probands of Belgian and Canadian UC trios is closer to 25 years old, the average age is approximately 40 for the Liege and Scottish UC cases (Table 5). Unlike CD where distinct categories of age of onset have been established by the Montreal classification (i.e. below 16, between 17–40, and above 40 years old) [36], such standards do not currently exist for UC. It is possible that UC patients with later age of onset have different risk factors involved than those individuals with earlier disease presentation. Such a difference in patients' age of onset could perhaps explain the lack of replication. Other differences in clinical subtype distribution in each case group, both UC and CD, may have also contributed to the lack of replication; unfortunately, no definitive conclusions may be reached since detailed information for some of the Centers that provided the samples is incomplete (Table 5).

**Table 5:** Demographic and clinical characteristics.

	Belgian CD Trios	Canadian CD Trios	Liege CD Cases^1^	Belgian UC Trios	Canadian UC Trios	Liege UC Cases^1^	Scottish UC Cases	Scottish Healthy Controls
Total Number of individuals	440	256	239	137	91	96	495	370
**Demographic**								
Gender								
Male	176 (40)	114 (47.9)	83 (34.7)	64 (46.7)	36 (45.6)	62 (64.6)	246 (51.7)	137 (50.2)
Female	264 (60)	124 (52.1)	156 (65.3)	73 (53.3)	43 (54.4)	34 (35.4)	230 (48.3)	136 (49.8)
**Clinical**								
Age at diagnosis mean±s.d. (range)	25.0±7.67 (10–50)	20.64±9.93 (2–43)	31.93±13.79 (3–75)	28.82±9.41 (11–58)	22.94±11.67 (3–54)	40.23±15.07 (5–72)	38.28±16.37 (9–81)	-
Smoking (%)	190 (48.1)	-	112 (49.3)	17 (16.5)	-	31 (32.6)	231 (50.4)	-
Unavailable data	45	-	12	34	-	1	37	-
Surgery (%)	213 (51.1)	-	103 (43.1)	24 (20.2)	-	9 (9.4)	87 (18.5)	-
Unavailable data	23	-	-	18	-	-	25	-
**Type of CD (%)**								
1- Inflammatory (non-stricturing, non-penetrating)	140 (51.9)	-	124 (54.1)	-	-	-	-	-
2- Stricturing	51 (18.9)	-	59 (25.8)	-	-	-	-	-
3- Penetrating	79 (29.3)	-	46 (20.1)	-	-	-	-	-
4- Unavailable data	170	-	10	-	-	-	-	-
**Extent CD (%)**								
1- Terminal ileum	90 (30.0)	-	94 (39.3)	-	-	-	-	-
2- Colon	129 (43.0)	-	86 (36.0)	-	-	-	-	-
3- Ileocolon	48 (16.0)	-	47 (19.7)	-	-	-	-	-
4- Upper GI	33 (11.0)	-	12 (5.0)	-	-	-	-	-
5- Unavailable data	140	-	-	-	-	-	-	-
**Extent UC (%)**								
1- Ulcerative proctitis	-	-	-	28 (23.7)	-	19 (20.7)	-	-
2- Left sided UC (distal UC)	-	-	-	39 (33.1)	-	38 (41.3)	-	-
3- Extensive UC (pancolitis)	-	-	-	51 (43.2)	-	35 (38.0)	-	-
4- Unavailable data	-	-	-	19	-	4	-	-
**Extra-intestinal manifestation (%)**	102 (38.9)	-	57 (28.1)	11 (21.2)	-	14 (17.1)	102 (28.6)	-
Unavailable data	178	-	36	85	-	14	138	-

1 Phenotypic information is not available for healthy controls from Liège.

This is also the first report evaluating the six *NLRP3* variants associated with CD [3] in a UC sample set. Interestingly, as is the case for the *NOD2* mutations associated uniquely to CD [37], no associations between the *NLRP3* variants and UC were observed. Given the similarities between *NOD2* and *NLRP3*, both members of the CATERPILLER family [10], and the above results, we conclude that the *NLRP3* association is likely to be specific to CD. Both gene products of *NOD2* and *NLRP3* play central roles in intracellular bacterial sensing and the CD associated variants of both genes were reported to result in a loss-of-function phenotype characterized by a decrease in secretion of IL-1β [3], [38]–[41]. Interestingly, the autophagy genes *IRGM* and *ATG16L1* have also been associated specifically to CD [42]–[43]. These results suggest that alterations in the intracellular sensing and processing of bacteria may constitute a central feature specific to the pathogenesis of CD, and that such primary defect in CD could result in the compensatory increase in the activity of Th1 cells that is observed in the lamina propria of patients with CD, but not in those with UC [37], [44]–[45]. We conclude that two of the key regulators of the inflammasomes, *NLRP3* and *MEFV*, do not seem to contribute to UC susceptibility.

Several factors prompted us to hypothesize that *MEFV* could equally well be implicated in CD and UC susceptibility: mutations in both *MEFV* and *NLRP3* cause auto-inflammatory diseases, these two genes encode for pyrin domain containing-proteins [10] that participate in the same shared signaling pathway (i.e. the inflammasome) [12]–[18], and the recent description of common variants in the *NLRP3* region associated with CD [3]. However, our results suggests otherwise since we observed no consistent associations with common variants in the *MEFV* region across all our screened sample sets and no epistatic interaction between *MEFV* and *NLRP3* variants. We thus conclude that variants in the *MEFV* region are unlikely to contribute to CD and UC susceptibility.

## Materials and Methods

### Ethics statement

All research involving mice were handled according to institutionally recommended animal care guidelines and all experiments were approved by the Animal Studies Ethics Committee of McGill University. The McGill University Health Centre Institutional Review Board approved the human expression study of colonic biopsy specimens and written informed consent was obtained from all participating subjects. The local Institutional Review Board of each Institution that sent DNA samples approved the human genetic study, and written informed consent was obtained from all participating subjects.

### Tissue collection

#### Animal experiments

Male BALB/c mice (between 6 and 8 weeks of age), obtained from Charles River Laboratory (St-Constant, Québec, Canada), were maintained under conventional housing conditions. Colitis was either induced by rectal instillation of 2.5 mg of trinitrobenzene sulfonic acid (TNBS) (Sigma Aldrich Canada Ltd., Ontario, Canada) or mice were fed with 5% dextran sulfate sodium (DSS) (MP Biomedicals, OH, USA) using modified protocols previously reported [46]–[47]. Human tissue biopsies: Colonic biopsy specimens were obtained from CD patients in ulcerated (severe involvement) and non-ulcerated (mild involvement) mucosa, from UC patients in inflamed areas as defined by Mayo sub-endoscopic criteria, and from patients who underwent colorectal cancer screening (CD n = 16; UC n = 17; Controls n = 25).

### RNA extraction and real-time quantitative PCR

Biopsies preserved in RNALater (Qiagen, Ontario, Canada) were homogenized and total RNA was extracted using TRIZOL (Invitrogen, Ontario, Canada). First strand cDNA was synthesized using the cDNA Archive Kit (Applied Biosystems (ABI), CA, USA) with MultiScribe reverse transcriptase and random primers. For both human and mice, primers and probes were ordered from Assay-on-demand ABI catalogue (mice *Mefv*: Mm00490260_gl; human *MEFV*: Hs00165145_ml; *18S*: 4319413E). Quantitative real-time PCR was performed using ABI prism 7900 sequence detection system based on the 5′ nuclease assay [48], and quantified using ABI's comparative Ct method. Statistical analyses were performed using the Wilcoxon Signed Ranks test to evaluate tissue (mice and human) expression differences (SPSS, Version 11.5, USA).

### Study populations

Three main sample sets, coming from 5 different Centers, were analyzed in this study (see detailed descriptions in Tables 1 and 5), comprising a total of 696 CD trios, 228 UC trios, 239 CD cases, 591 UC cases, and 477 controls. All patients were recruited through specialized hospitals, academic centres, and practitioners. Inflammatory bowel disease (IBD) specialists involved in this study confirmed the diagnosis of CD and UC using standard criteria further described below [36], [49], and patients were excluded from the study in the case of doubtful diagnosis. In all the participating Centers, the diagnosis of IBD was made after fulfilling standard clinical, radiological, endoscopic, and pathology criteria [1], [36], [49] that required (1) one or more of the following symptoms: diarrhea, rectal bleeding, abdominal pain, weight loss, fever or complicated perianal disease; (2) occurrence of symptoms on two or more occasions in the past or ongoing symptoms of at least 4–6 weeks' duration; (3) evidence of inflammation, strictures or fistula from radiological, endoscopic, and histological evaluation (with some specific CD characteristics); (4) exclusion of all other diagnosis besides CD.

Belgian subjects (referred to as the combined Belgian cohort, Table 1), all of European descent, were used for the exploratory phase of the study and these came from Center 1 (University of Leuven, Belgium) and Center 2 (University of Liege, Belgium). The replication cohorts consisted of Canadian subjects (referred to as the combined Canadian cohort, Table 1), coming from Centers 3 and 4, and Scottish subjects from Edinburgh (Centre 5, Table 1). Center 3 is composed of subjects collected from multiple sites in the province of Québec (Canada), which are all of European ancestry, and includes 34 probands of Ashkenazi Jewish ancestry and 6 of Sephardic Jewish ancestry. Center 4 includes subjects collected from multiple sites in Toronto (Canada). The vast majority of probands are of European ancestry, including 34 probands of Ashkenazi Jewish ancestry, and 15 probands are of non-European ancestry. Finally, cases and matched controls from Center 5 were collected from multiple sites in Edinburgh (Scotland). The majority of cases are of European ancestry, including 1 case of Jewish ancestry, except for 14 non-European cases and 11 non-European controls. Center 5 will be referred to as the Scottish cohort. Overall, only samples of European origin were included in the analysis.

### Genotyping experiments

Single nucleotide polymorphism (SNP) genotyping was performed using either the GenomeLab SNPstream Genotyping System (Beckman Coulter, CA, USA) [50], the fluorescence polarization template-directed dye-terminator (FP-TDI) system (PerkinElmer, MA, USA) [51], or the Taqman 5′ exonuclease assay (ABI, CA, USA) (rs2242217: assay C_2394717_20) [52]. Also, since two non-synonymous variants (rs3743930, rs224222) as well as three synonymous variants (rs224225, rs224224, rs224223) were observed in *MEFV* exon 2 during the sequencing screening phase, genotyping of these variants was done using a sequencing approach. All primers and probes are available in Table S4.

We used 4 quality checks to determine whether SNPs were reliable and informative: (1) genotyping efficiency (>95%), (2) compliance to Hardy-Weinberg equilibrium (p>0.01), (3) Mendelian inheritance, and (4) population minor allele frequency (>5%).

### Sequencing

Sequencing was performed on an ABI 3730 DNA Sequencer according to standard protocols. Sequence traces were assembled and analyzed using a modified version of the PolyPhred software package. Primer sequences are available in Table S5. The sequences were compared to the annotated sequences (NCBI Build 36.1, hg18) of 25 exons and promoter regions of *MEFV*, *AK096958*, *ZNF263*, *TIGD7* and *ZNF75A* to identify novel variants.

### Statistical analysis

To evaluate *MEFV* as a candidate gene for IBD, we divided the study into an exploratory and replication phase. Mendelian errors and departures from Hardy-Weinberg equilibrium were assessed using MERLIN v0.9.10. Measures of pairwise LD between SNPs (*D'* and *r^2^*) were computed using Haploview v.4.1 [53]. Tests of association were performed using the likelihood methods implemented in UNPHASED v3.0.10 [54], which can analyze samples of nuclear families, unrelated subjects, or a combination of both. It also allows tests to be conditional on each individual's genotype at one marker to test for association at another, to look for associations that may have been missed due to the possibility of epistasis between two loci. It also tests for the presence of gene-gene interaction (i.e. merely, the departure from log-additive effects of two or more SNPs on the risk). Analysis was done using the default settings; adjustment for Study Center in the combined analysis was done using the options –confounder and –factor; conditional analyses were done using the options –condition and –condgenotype, and gene-by-gene interactions was tested by adding the option –model gxg. To evaluate the significance of our results after correcting for the number of SNPs tested, we used the options –permutation 1000 and –permoutput, and corrected further for the two diseases tested.

## Supporting Information

Figure S1 *Linkage disequilibrium structure between tagging SNPs and MEFV exon 2 coding SNPs screened in the combined Belgian IBD sample set.* Shown above are the SNPs with their positions in the genes and the LD structure between them. SNPs in red are exonic. The upper left portion of the coloured matrix is *D'* and the lower right portion is *r^2^*. Data for the first SNP is represented on the left column and the bottom row of the matrix.
(1.61 MB PSD)

Figure S2 *Conditional tests of association and tests of gene-gene interactions between SNPs in MEFV* and in *NLRP3* in the combined Belgian-Canadian CD sample set. *P values* for the tests are color coded and represented. For each combination of SNPs, the upper triangular portions of each square represent the *p* value for testing the association of the *MEFV* SNP (horizontal axis) conditional on each individual's genotype at the *NLRP3* SNP (vertical axis). The lower triangular portions of each square represent the *p* value for the test of statistical interaction between the two SNPs. The unconditional tests of association for each SNP in *MEFV* are shown on the line labeled “none”. The two *MEFV* non-synonymous variants *C310S* and *R202Q* are also referred to as rs220379 and rs224222, respectively.
(0.91 MB TIF)

Figure S3 *Conditional tests of association and tests of gene-gene interactions between SNPs in MEFV* and in *NLRP3* in the combined Belgian-Canadian UC sample set. Refer to legend of Figure S2.
(0.95 MB TIF)

Table S1 *Exploratory phase association results of the SNP panel genotyped in the combined Belgian CD and UC trios sample sets.*
(0.11 MB DOC)

Table S2 *Rare variants uncovered in MEFV* exon 2.
(0.07 MB DOC)

Table S3 *Association results of NLRP3* tagging SNPs in UC sample sets.
(0.04 MB DOC)

Table S4 *List of oligos and probes used to perform the genotyping experiments.*
(0.15 MB PDF)

Table S5 *List of oligos used to amplify fragments for the sequencing experiments.*
(0.14 MB DOC)
