# Supplementary figures and images for: Genetic Variation in the Familial Mediterranean Fever Gene (MEFV) and Risk for Crohn's Disease and Ulcerative Colitis
Source: PLoS One. 2009 Sep 28;4(9):e7154. doi: 10.1371/journal.pone.0007154 (PMC2745755; doi:10.1371/journal.pone.0007154)

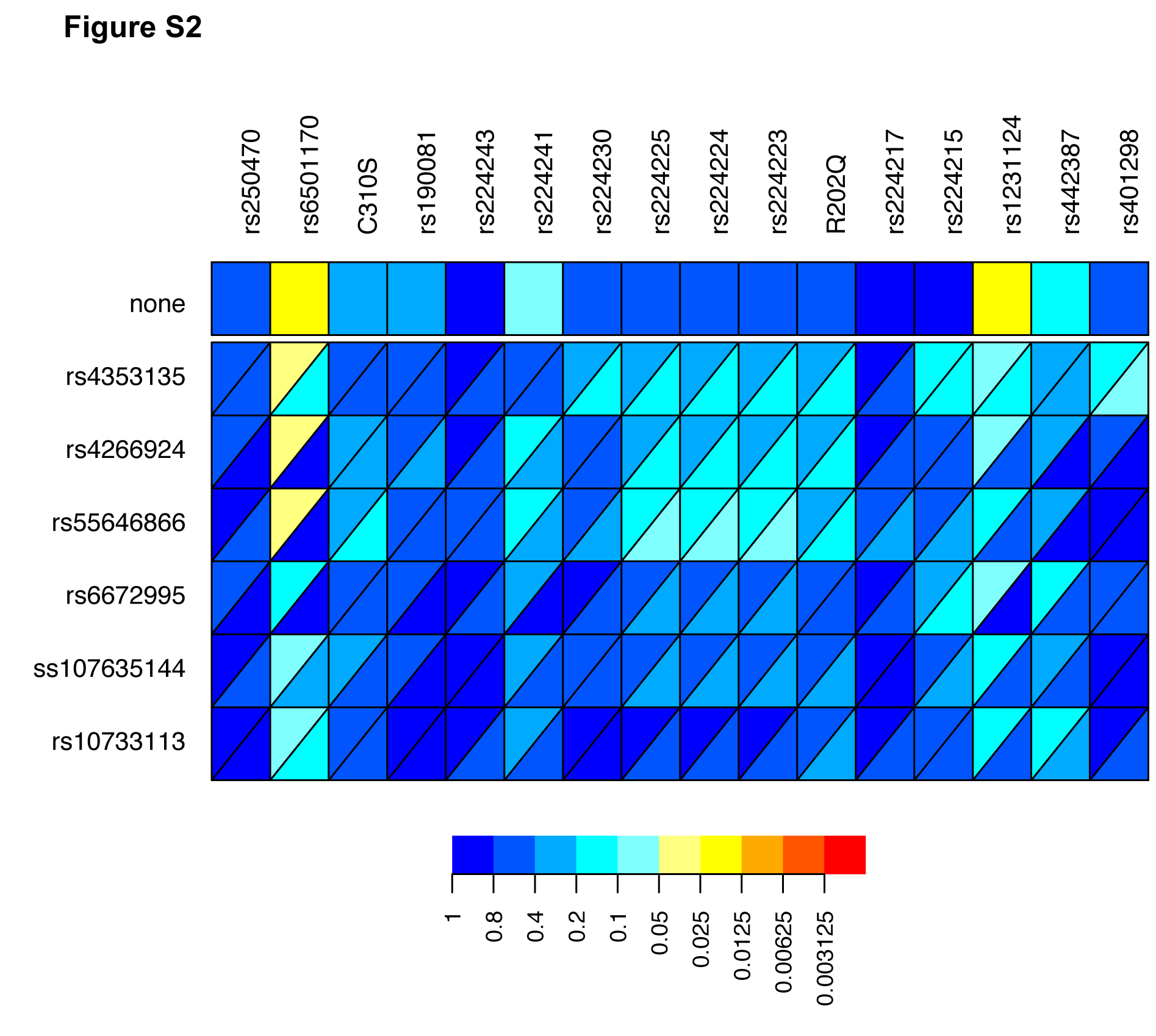

Supplement: Figure S2 — Conditional tests of association and tests of gene-gene interactions between SNPs in MEFV and in NLRP3 in the combined Belgian-Canadian CD sample set. P values for the tests are color coded and represented. For each combination of SNPs, the upper triangular portions of each square represent the p value for testing the association of the MEFV SNP (horizontal axis) conditional on each individual's genotype at the NLRP3 SNP (vertical axis). The lower triangular portions of each square represent the p value for the test of statistical interaction between the two SNPs. The unconditional tests of association for each SNP in MEFV are shown on the line labeled “none”. The two MEFV non-synonymous variants C310S and R202Q are also referred to as rs220379 and rs224222, respectively. (0.91 MB TIF) [file pone.0007154.s002.tif]

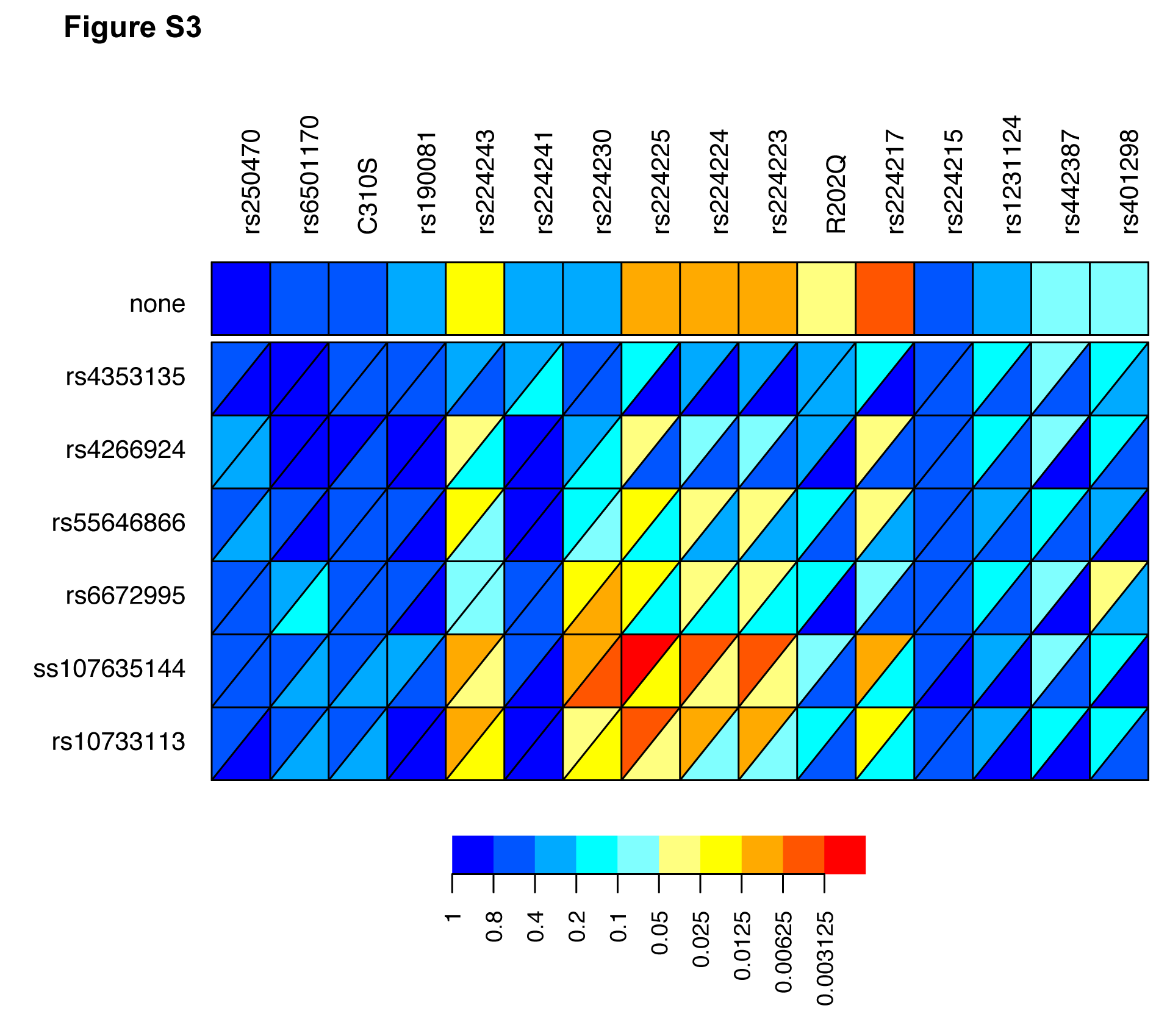

Supplement: Figure S3 — Conditional tests of association and tests of gene-gene interactions between SNPs in MEFV and in NLRP3 in the combined Belgian-Canadian UC sample set. Refer to legend of Figure S2. (0.95 MB TIF) [file pone.0007154.s003.tif]
