# Supplementary material for: Genetic Variation in the Familial Mediterranean Fever Gene (MEFV) and Risk for Crohn's Disease and Ulcerative Colitis
Source: PLoS One. 2009 Sep 28;4(9):e7154. doi: 10.1371/journal.pone.0007154 (PMC2745755; doi:10.1371/journal.pone.0007154)
Supplement: Table S1 — Exploratory phase association results of the SNP panel genotyped in the combined Belgian CD and UC trios sample sets. (0.11 MB DOC) [file pone.0007154.s004.doc]

**Table S1:** *Exploratory phase association results of the SNP panel genotyped in the combined Belgian CD and UC trios sample sets.*

|  |  |  | **Belgian CD^1^** | | | | **Belgian UC^2^** | | | |
| --- | --- | --- | --- | --- | --- | --- | --- | --- | --- | --- |
| **#** | **SNP ID** | **Position dbSNP130** | **Allele^3^** | **Frequency Cases** | **Frequency Controls** | ***P* value** | **Allele^3^** | **Frequency Cases** | **Frequency Controls** | ***P* value** |
| 1 | rs250470 | 3318978 | T | 0.27 | 0.25 | 0.2995 | C | 0.79 | 0.74 | 0.3446 |
| 2 | rs6501170 | 3317583 | T | 0.87 | 0.82 | ***0.0155*** | T | 0.81 | 0.79 | 0.6681 |
| 3 | rs7500738 | 3302610 | C | 0.24 | 0.24 | 0.8989 | C | 0.27 | 0.24 | 0.5285 |
| 4 | rs757108 | 3302018 | T | 0.76 | 0.76 | 0.9491 | C | 0.27 | 0.24 | 0.6113 |
| 5 | rs1149483 | 3296835 | T | 0.58 | 0.53 | 0.0594 | C | 0.55 | 0.40 | ***0.0059*** |
| 6 | rs6501169 | 3293206 | T | 0.77 | 0.76 | 0.5335 | C | 0.26 | 0.23 | 0.4919 |
| 7 | rs8052682 | 3287034 | T | 0.76 | 0.76 | 0.8951 | C | 0.26 | 0.24 | 0.6961 |
| 8 | rs220379/C310S | 3279436 | G | 0.76 | 0.76 | 0.7928 | C | 0.27 | 0.22 | 0.3446 |
| 9 | rs188760 | 3276562 | T | 0.24 | 0.24 | 0.8468 | T | 0.26 | 0.24 | 0.6054 |
| 10 | rs458522 | 3270180 | T | 0.24 | 0.24 | 0.9491 | T | 0.27 | 0.23 | 0.4382 |
| 11 | rs190081 | 3263570 | C | 0.24 | 0.23 | 0.7946 | C | 0.27 | 0.24 | 0.5148 |
| 12 | rs224243 | 3259194 | C | 0.57 | 0.52 | 0.0920 | T | 0.56 | 0.40 | ***0.004553*** |
| 13 | rs224241 | 3257037 | C | 0.22 | 0.22 | 1.0000 | C | 0.29 | 0.18 | 0.0840 |
| 14 | rs224231 | 3249980 | G | 0.60 | 0.54 | ***0.0394*** | A | 0.57 | 0.42 | ***0.0308*** |
| 15 | rs224230 | 3248358 | G | 0.35 | 0.34 | 0.6872 | A | 0.72 | 0.59 | ***0.0356*** |
| 16 | rs224226 | 3245733 | A | 0.57 | 0.52 | 0.0592 | G | 0.56 | 0.38 | ***0.002867*** |
| 17 | rs224225 | 3244763 | T | 0.53 | 0.51 | 0.3365 | C | 0.58 | 0.40 | ***0.00082*** |
| 18 | rs182674 | 3243311 | A | 0.56 | 0.50 | ***0.0294*** | G | 0.57 | 0.36 | ***0.001159*** |
| 19 | rs224217 | 3241758 | G | 0.56 | 0.51 | 0.0524 | A | 0.58 | 0.39 | ***0.000689*** |
| 20 | rs224215 | 3241361 | A | 0.64 | 0.64 | 0.9512 | G | 0.42 | 0.29 | 0.0508 |
| 21 | rs224213 | 3239750 | C | 0.48 | 0.45 | 0.3673 | C | 0.50 | 0.49 | 0.8981 |
| 22 | rs224212 | 3238866 | A | 0.47 | 0.45 | 0.6949 | G | 0.50 | 0.48 | 0.7728 |
| 23 | rs224208 | 3237182 | G | 0.47 | 0.46 | 0.8033 | G | 0.52 | 0.51 | 0.8886 |
| 24 | rs1231124 | 3234679 | A | 0.47 | 0.46 | 0.8510 | A | 0.52 | 0.48 | 0.5860 |
| 25 | rs2741919 | 3232897 | C | 0.49 | 0.43 | 0.0752 | T | 0.51 | 0.45 | 0.4227 |
| 26 | rs71999 | 3231468 | A | 0.48 | 0.45 | 0.2509 | G | 0.50 | 0.48 | 0.7773 |
| 27 | rs8054015 | 3228830 | G | 0.58 | 0.57 | 0.7987 | A | 0.45 | 0.42 | 0.7054 |
| 28 | rs442387 | 3226119 | G | 0.57 | 0.52 | 0.1011 | A | 0.43 | 0.42 | 0.8886 |
| 29 | rs2075852 | 3222606 | T | 0.56 | 0.51 | 0.1379 | C | 0.44 | 0.40 | 0.5462 |
| 30 | rs401298 | 3220975 | A | 0.43 | 0.41 | 0.5520 | A | 0.48 | 0.45 | 0.6616 |

^1^Includes 440 CD trios.

^2^Includes 137 UC trios.

^3^Alleles shown are the alleles seen more frequently in the cases than in the controls.
