# Supplementary material for: Genetic Variation in the Familial Mediterranean Fever Gene (MEFV) and Risk for Crohn's Disease and Ulcerative Colitis
Source: PLoS One. 2009 Sep 28;4(9):e7154. doi: 10.1371/journal.pone.0007154 (PMC2745755; doi:10.1371/journal.pone.0007154)
Supplement: Table S2 — Rare variants uncovered in MEFV exon 2. (0.07 MB DOC) [file pone.0007154.s005.doc]

**Table S2:** *Rare variants uncovered in MEFV exon 2*

| **Type of Cohort** | **Base Change^1^** | **Amino Acid Change** | **Located on CpG dinucleotide** | **Disease Subgroup** | **Cohorts^2^** | **# Times Transmitted** |
| --- | --- | --- | --- | --- | --- | --- |
| CD Trios | c.331G>A | G111R | no | mother and its CD case | Belgian Trios | 1 |
|  | c.372C>T | P124P | yes | mother and its CD case | Canadian Trios | 1 |
|  | c.406G>T | G136W | no | father and its CD case | Belgian Trios | 1 |
|  | c.408G>A | G136G | no | mother and its CD case | Belgian Trios | 1 |
|  | c.608T>C | L203P | no | affected mother of CD case | Canadian Trios | 0 |
|  | c.656G>A | G219E | no | father of CD case | Belgian Trios | 0 |
|  | c.702C>T | P234P | no | mother of CD case | Canadian Trios | 0 |
| UC Trios | c.297C>T | N99N | yes | mother and its UC case | Canadian Trios | 1 |
|  | c.388A>G | N130S | no | mother and its UC case | Belgain Trios | 1 |
|  | c.578C>T | A193V | yes | mother and its UC case;  father and its UC case | Belgian Trios | 2 |
|  | c.617A>G | N206S | no | mother of UC case | Belgian Trios | 0 |
|  | c.656G>A | G219E | no | mother and its UC cases | Belgian Trios | 1 |
|  | c.664G>A | G222R | no | mother and its UC case | Belgian Trios | 1 |
|  | c.717A>T | R239R | no | mother and its UC case | Belgian Trios | 1 |
| CD sporadic | c.653G>C | G218A | no | CD case | Liege C/C | - |
| UC sporadic | c.289C>A | Q97K | no | UC case | Scottish C/C | - |
|  | c.498G>C | S166S | no | UC case | Scottish C/C | - |
|  | c.505C>G | L169V | no | UC case | Scottish C/C | - |
|  | c.549G>A | P183P | no | UC case | Scottish C/C | - |
|  | c.657C>T | G219G | yes | UC case | Scottish C/C | - |
| Unaffected | c.388A>G | N130S | no | unaffected controls | Scottish C/C | - |
|  | c.476G>A | S159N | no | unaffected control | Liege C/C | - |
|  | c.493G>A | A165T | no | unaffected control | Scottish C/C | - |

^1^ According to the cDNA coding sequence, with +1 from the A of the initiating ATG. Reference sequence is NM_000243 (*MEFV*).

^2^ C/C refers to case control sample set.
