# Supplementary material for: Genetic Variation in the Familial Mediterranean Fever Gene (MEFV) and Risk for Crohn's Disease and Ulcerative Colitis
Source: PLoS One. 2009 Sep 28;4(9):e7154. doi: 10.1371/journal.pone.0007154 (PMC2745755; doi:10.1371/journal.pone.0007154)
Supplement: Table S3 — Association results of NLRP3 tagging SNPs in UC sample sets. (0.04 MB DOC) [file pone.0007154.s006.doc]

**Table S3:** Association results of *NLRP3* tagging SNPs in UC sample sets

|  | **Combined Belgian UC^1^** | | | | **Combined Canadian UC^2^** | | | | **Combined UC^3^** | | | |
| --- | --- | --- | --- | --- | --- | --- | --- | --- | --- | --- | --- | --- |
| **SNP** | **Allele^4^** | **Frequency Cases** | **Frequency Controls** | ***P* value** | **Allele^4^** | **Frequency Cases** | **Frequency Controls** | ***P* value** | **Allele^4^** | **Frequency Cases** | **Frequency Controls** | ***P* value** |
| rs4353135 | T | 0.67 | 0.66 | 0.7506 | G | 0.34 | 0.34 | 0.8907 | T | 0.67 | 0.66 | 0.7654 |
| rs4266924 | A | 0.87 | 0.81 | 0.3767 | A | 0.89 | 0.86 | 0.3345 | A | 0.88 | 0.82 | 0.3806 |
| rs55646866 | C | 0.89 | 0.83 | 0.4734 | C | 0.90 | 0.85 | 0.2182 | C | 0.89 | 0.84 | 0.3046 |
| rs6672995 | G | 0.82 | 0.79 | 0.9175 | A | 0.17 | 0.17 | 1.0000 | G | 0.83 | 0.80 | 0.9426 |
| ss107635144 | C | 0.85 | 0.79 | 0.3470 | C | 0.86 | 0.83 | 0.4645 | C | 0.85 | 0.80 | 0.3466 |
| rs10733113 | G | 0.85 | 0.80 | 0.5143 | G | 0.86 | 0.83 | 0.6013 | G | 0.85 | 0.81 | 0.5195 |

^1^Includes 137 UC trios, 96 UC cases, and 107 healthy controls.

^2^Includes 91 UC trios.

^3^Includes 228 UC trios, 96 UC cases, and 107 healthy controls.

^4^Alleles shown are the alleles seen more frequently in the cases than in the controls.
