# Supplementary material for: Genetic Variation in the Familial Mediterranean Fever Gene (MEFV) and Risk for Crohn's Disease and Ulcerative Colitis
Source: PLoS One. 2009 Sep 28;4(9):e7154. doi: 10.1371/journal.pone.0007154 (PMC2745755; doi:10.1371/journal.pone.0007154)
Supplement: Table S4 — List of oligos and probes used to perform the genotyping experiments. (0.15 MB PDF) [file pone.0007154.s007.pdf]

Table S4: *List of oligos and probes used to perform the genotyping experiments*

Oligo ID	Primers	Probes	Genotyping Technology	Comments
rs11466023_P369S_F	YGGTGCCAGGACTCCCAT		SNPstream panel I	Below 5% minor allele frequency
rs11466023_P369S_R	TGGACCTGCTTCAGGTGG		SNPstream panel I	Below 5% minor allele frequency
rs11466023_P369S_CT_U1	-	ACGCACGTCCACGGTGATTTAAGGAAGAGCCCGGGAAGCCTAAGC	SNPstream panel I	Below 5% minor allele frequency
rs7187449_F	TCCTGGAACATGGAATGG	-	SNPstream panel I	Monomorphic marker
rs7187449_R	AAGGCAGCATGTAGGGGT	-	SNPstream panel I	Monomorphic marker
rs7187449_CT_U2	-	GGATGGCGTTCCGTCCTATTTCTTTATACAGACAACTAAGACTGG	SNPstream panel I	Monomorphic marker
rs224225_D102D_F	TTCATCATTTTGCATCTGGTT	-	SNPstream panel I	-
rs224225_D102D_R	TCTTCAGGCTCCTGGGCT	-	SNPstream panel I	-
rs224225_D102D_CT_U2	-	GGATGGCGTTCCGTCCTATTATTCCACACAAGAAAACGGCACAGA	SNPstream panel I	-
rs224241_F	AAATACCACCTGATGTTGGCT	-	SNPstream panel I	-
rs224241_R	TGAAATTTTAAAAATGCAGTTGAG	-	SNPstream panel I	-
rs224241_CT_U3	-	CGTGCCGCTCGTGATAGAATAAGAACCAAAGCTTTTCTTGGCAAA	SNPstream panel I	-
rs61754767_R42W_F	TTCAAGTTCAAGCTGCAGAAC	-	SNPstream panel I	Below 5% minor allele frequency
rs61754767_R42W_R	AGAGTGGCCATCTTCACC	-	SNPstream panel I	Below 5% minor allele frequency
rs61754767_R42W_CT_U4	-	AGCGATCTGCGAGACCGTATGCAGAAGGAGCACTCCAGGATCCCC	SNPstream panel I	Below 5% minor allele frequency
rs28940579_ V726A_F	TAATAAAGGAGCCTCCCAAGC	-	SNPstream panel I	Below 5% minor allele frequency
rs28940579_ V726A_R	CCAGAGAAAGAGCAGCTGG	-	SNPstream panel I	Below 5% minor allele frequency
rs28940579_V726A_CT_U5	-	GCGGTAGGTTCCCGACATATGTGGGCATCTTCGTGGACTACAGAG	SNPstream panel I	Below 5% minor allele frequency
rs2075852_F	AAATACATTCAAATCCTCAAAGACC	-	SNPstream panel I	-
rs2075852_R	ATTTATAGGTCCCTCTTGCTATCC	-	SNPstream panel I	-
rs2075852_CT_U6	-	GGCTATGATTCGCAATGCTTAACTGAGGTTCACTGACAGCCCTAC	SNPstream panel I	-
rs8052921_F	GTGCTGTGTGATCCCATCTC	-	SNPstream panel I	Marker did not work
rs8052921_R	TCTGTTGCAAAACGTGTCAC	-	SNPstream panel I	Marker did not work
rs8052921_CT_U7	-	AGGGTCTCTACGCTGACGATTGGTCAGAAAACTCCTCGGAGCTGA	SNPstream panel I	Marker did not work
rs11466018_L110PCT_F	AATATTCCACACAAGAAAACGG	-	SNPstream panel I	Monomorphic marker
rs11466018_L110PCT_R	RGGGTGGTCTGGAGTCTTC	-	SNPstream panel I	Monomorphic marker
rs11466018_L110PCT_U8	-	GTGATTCTGTACGTGTCGCCGAYGAYTCCGCAGCGTCCAGMTCCC	SNPstream panel I	Monomorphic marker

rs2741919_F	TTCAGGAGCACCTGAGAGTG	-	SNPstream panel I	-
rs2741919_R	ACTGATGACACACCATGGATT	-	SNPstream panel I	-
rs2741919_CT_U8	-	GTGATTCTGTACGTGTCGCCCACYCACCAGGGGCGGATTATGCAA	SNPstream panel I	-
rs11466045_I591T_F	TTCCTTGTCTTTCCTTGTTGTC	-	SNPstream panel I	Below 5% minor allele frequency
rs11466045_I591T_R	ACTTGCCTTGATCTGGGC	-	SNPstream panel I	Below 5% minor allele frequency
rs11466045_I591T_CT_U9	-	GACCTGGGTGTCGATACCTATTTTTCTCCGTAGTTCCRGAGCTGA	SNPstream panel I	Below 5% minor allele frequency
rs417929_F	AAAGTGGGGTGTGGCAGG	-	SNPstream panel I	Monomorphic marker
rs417929_R	CTTTATTTTTTACAAACCCTCTTGTT	-	SNPstream panel I	Monomorphic marker
rs417929_CT_U10	-	AGATAGAGTCGATGCCAGCTTAACCCTGCTGAAGGGCTGCAGTCA	SNPstream panel I	Monomorphic marker
rs224243_F	AATATGATTCCTTTCGGATAACTTG	-	SNPstream panel I	-
rs224243_R	AGCAACATCCCGGTTGTC	-	SNPstream panel I	-
rs224243_CT_U10	-	AGATAGAGTCGATGCCAGCTGATGGAAAATCACAGGCCAAAGTCA	SNPstream panel I	-
rs1149487_F	ATCTTCATGTGTGGGCACC	-	SNPstream panel I	Marker did not work
rs1149487_R	ATTAAGAATTGTTTCCAGCTGC	-	SNPstream panel I	Marker did not work
rs1149487_CT_U11	-	AGAGCGAGTGACGCATACTAGCTGCGGCTGGAACCACAGTGGACA	SNPstream panel I	Marker did not work
T267I_F	AACTTTAATATCCAAGGGGATTC	-	SNPstream panel I	Below 5% minor allele frequency
T267I_R	TTCTCTGCAGCCGATATAAAGTA	-	SNPstream panel I	Below 5% minor allele frequency
T267I_CT_U12	-	CGACTGTAGGTGCGTAACTCATTCTCCTGACTCTAGAGGAAAAGA	SNPstream panel I	Below 5% minor allele frequency
rs401298_F	AAATCTTACCTCAGTCTTGCGA	-	SNPstream panel II	-
rs401298_R	TTCTGGAATTGTATTCATCAGGT	-	SNPstream panel II	-
rs401298_GA_U1	-	ACGCACGTCCACGGTGATTTGTCTTACAAACCAAATTGGAAG	SNPstream panel II	-
rs224230_F	AAGAATCTGTAGCTTAGTGACTTGC	-	SNPstream panel II	-
rs224230_R	TCAGTGGTCAGCTGGAAAT	-	SNPstream panel II	-
rs224230_GA_U2	-	GGATGGCGTTCCGTCCTATTCATGACAATAATTTGTGATTTTACT	SNPstream panel II	-
rs8054015_F	TCATGATATGTTTATACAGTGGAAG	-	SNPstream panel II	-
rs8054015_R	TTATAACTTGCCTTTTCACTTAAAGTATC	-	SNPstream panel II	-
rs8054015_GA_U3	-	CGTGCCGCTCGTGATAGAATATATGTTTATACAGTGGAAGCCAAC	SNPstream panel II	-
rs224217_F	ATATATGCACAGCTTCACAAATGT	-	SNPstream panel II	-
rs224217_R	TGTGAGGTTATTGTGAGAGGG	-	SNPstream panel II	-
rs224217_GA_U4	-	AGCGATCTGCGAGACCGTATGTCTTCCCCAGATTTTCTACCTGGT	SNPstream panel II	-
rs71999_F	AAACTCCTATGCCAACTCCT	-	SNPstream panel II	-

rs71999_R	GCAAGTTTACCTGCTCTCATC	-	SNPstream panel II	-
rs71999_GA_U5	-	GCGGTAGGTTCCCGACATATTGTCTTCACTACTAGAGTGTAGATT	SNPstream panel II	-
rs11466024_R408Q_F	CYATCTGCCTCATCTGCAG	-	SNPstream panel II	Below 5% minor allele frequency
rs11466024_R408Q_R	TGCCTACCTTGTGTTCCAG	-	SNPstream panel II	Below 5% minor allele frequency
rs11466024_R408Q_GA_U6	-	GGCTATGATTCGCAATGCTTCTGAGTCAGGAGCACCAAGGCCACC	SNPstream panel II	Below 5% minor allele frequency
rs224208_F	AAACAAACTGAAGCGCTGAA	-	SNPstream panel II	-
rs224208_R	ATCTGGCCCACGTCCTCC	-	SNPstream panel II	-
rs224208_GA_U7	-	AGGGTCTCTACGCTGACGATTGGAGCAGGTGTACTACTTCCTGGA	SNPstream panel II	-
rs224212_F	TTTCTGGTAAGGTCAGAGGTG	-	SNPstream panel II	-
rs224212_R	AACCACAGCAGAATCTCGG	-	SNPstream panel II	-
rs224212_GA_U8	-	GTGATTCTGTACGTGTCGCCGATGGCCCATCCGTCCCTGGGAGGA	SNPstream panel II	-
rs224226_GA_F	TATAATCTGGGTTTTGCTGACC	-	SNPstream panel II	-
rs224226_GA_R	ATTATTTATAAGTGTGAGCCACCAT	-	SNPstream panel II	-
rs224226_GA_U8	-	GTGATTCTGTACGTGTCGCCCAGTTGTCCTGCTTAAAACGTTTGA	SNPstream panel II	-
rs224205_F	AGCCCTGGTAAGTGCAGC	-	SNPstream panel II	Marker did not work
rs224205_R	AAAAAAGAAGGAAACTGTCGGT	-	SNPstream panel II	Marker did not work
rs224205_GA_U9	-	GACCTGGGTGTCGATACCTAGAGCTAAAAGTCCAGGAGCCCAGAA	SNPstream panel II	Marker did not work
rs224231_GA_F	GGGCACGGGACTGATACTC	-	SNPstream panel II	-
rs224231_GA_R	ACGGCAAAACCTCGTCTCTA	-	SNPstream panel II	-
rs224231_GA_U9	-	GACCTGGGTGTCGATACCTAAACCTCCACCTCCCAGGTTCAAGCA	SNPstream panel II	-
rs1231124_F	GTGGCCCAAGTACCCGTG	-	SNPstream panel II	-
rs1231124_R	AAAGTTGAGGACCAGCATTTAG	-	SNPstream panel II	-
rs1231124_GA_U10	-	AGATAGAGTCGATGCCAGCTCTGGAAATGAACTACATTCTCCACA	SNPstream panel II	-
rs442387_F	TCTGGTGACTCCCATAGGT	-	SNPstream panel II	-
rs442387_R	AAACCGAGACAGTAGGAGAAGG	-	SNPstream panel II	-
rs442387_GA_U11	-	AGAGCGAGTGACGCATACTAGCAGTGTAGAAGTTAGCAAGCTGGA	SNPstream panel II	-
rs61752717_M694V_F	GAGGTGGAGGTTGGAGACAA	-	SNPstream panel II	Below 5% minor allele frequency
rs61752717_M694V_R	AGAGCAGCTGGCGAATGTAT	-	SNPstream panel II	Below 5% minor allele frequency
rs61752717_M694V_GA_U12	-	CGACTGTAGGTGCGTAACTCGAATGGCTACTGGGTGGTGATAATG	SNPstream panel II	Below 5% minor allele frequency
rs224215_GA_F	TCCCAGAAATTCACAGCACA	-	SNPstream panel II	-
rs224215_GA_R	GCAAGATGGAGGAACAGAGG	-	SNPstream panel II	-

rs224215_GA_U12	-	CGACTGTAGGTGCGTAACTCTTCTAGAGCTTTCCCGAGGGATACT	SNPstream panel II	-
rs1149483_F	GAGCATCTTGTTGCTTACTTGG	-	SNPstream panel III	-
rs1149483_R	GCAGTTGACAGCGGGTAAGT	-	SNPstream panel III	-
rs1149483_CT_U1	-	ACGCACGTCCACGGTGATTTGTCTGAAAATAGGCTACTTTTTTAC	SNPstream panel III	-
rs11645952_F	TCAGTCGAGCGTCTGTAAGG	-	SNPstream panel III	Below 5% minor allele frequency
rs11645952_R	TGTCAGCGCTAAGATTGGTG	-	SNPstream panel III	Below 5% minor allele frequency
rs11645952_CT_U2	-	GGATGGCGTTCCGTCCTATTTACGATTCACCCAAATCTGATAATG	SNPstream panel III	Below 5% minor allele frequency
rs6501170_F	CTCATTGAGAACACAGGAGCTG	-	SNPstream panel III	-
rs6501170_R	AGTATCCCTTCCGGACCACT	-	SNPstream panel III	-
rs6501170_CT_U3	-	CGTGCCGCTCGTGATAGAATAACACCAGGCATCCCAGATGCCTCG	SNPstream panel III	-
rs6501169_F	TCTCAGCTGTGCCGTCGT	-	SNPstream panel III	-
rs6501169_R	TGACAGGCCTGATTCATAACA	-	SNPstream panel III	-
rs6501169_CT_U4	-	AGCGATCTGCGAGACCGTATAAACCAAAAGGAAAGGCTCAAGGAA	SNPstream panel III	-
rs8052682_F	CCACTCTTGCTCTAGTACCTTGC	-	SNPstream panel III	-
rs8052682_R	TGTCTGGAGGAGGCTTGAAT	-	SNPstream panel III	-
rs8052682_CT_U5	-	GCGGTAGGTTCCCGACATATTGCTGCGATTACGGGTGTGAGCCAC	SNPstream panel III	-
rs250470_F	ACTTGCCTGGTGGAGAACAG	-	SNPstream panel III	-
rs250470_R	CTCCGTCAGTGAGCCAAGAT	-	SNPstream panel III	-
rs250470_CT_U6	-	GGCTATGATTCGCAATGCTTCAACACATGAAATAAAACAGTTGGG	SNPstream panel III	-
rs458522_F	GGCGCTTACTTCTCTTCACTACTT	-	SNPstream panel III	-
rs458522_R	CCAATGCAGTTCCAGAGGAT	-	SNPstream panel III	-
rs458522_CT_U7	-	AGGGTCTCTACGCTGACGATAAGTAGCAGTTCTTAGTCTTTTTGG	SNPstream panel III	-
rs220380_F	AGAGGAGCCTGTTGGCAAG	-	SNPstream panel III	Marker did not work
rs220380_R	GACAGTCAGGTGCCCAAATC	-	SNPstream panel III	Marker did not work
rs220380_CT_U8	-	GTGATTCTGTACGTGTCGCCTCCTACCCTCTTGGTCTTTTTAAAG	SNPstream panel III	Marker did not work
rs190081_F	AATGTCTCCATGCTCTACAGGTT	-	SNPstream panel III	-
rs190081_R	CAGTTGTTAAGAGTGGCAATGG	-	SNPstream panel III	-
rs190081_CT_U9	-	GACCTGGGTGTCGATACCTATGACATCTATCTAGGTCCAGATGAA	SNPstream panel III	-
rs188760_F	ACATGCAGTATTCCAGGATGC	-	SNPstream panel III	-
rs188760_R	TGTGAGAGATGATAGGCTGGAA	-	SNPstream panel III	-
rs188760_U10	-	AGATAGAGTCGATGCCAGCTTTCCGTTAGTGTTTCTAGGCTCGTC	SNPstream panel III	-

rs7500738_F	AAGAGACTGCTTCAGGCATCA	-	SNPstream panel III	-
rs7500738_R	CGTGCTCAGGTGTCACTGTC	-	SNPstream panel III	-
rs7500738_CT_U11	-	AGAGCGAGTGACGCATACTATCTGTACTTAAAAAGTCAGGATCAG	SNPstream panel III	-
rs757108_F	CAAGAACAGCTCAGCAGGAA	-	SNPstream panel III	-
rs757108_R	CTACTGCTGCCTGGAAGGAC	-	SNPstream panel III	-
rs757108_CT_U12	-	CGACTGTAGGTGCGTAACTCAATAGTTTATTCACCCAAAATGTCA	SNPstream panel III	-
rs224230b_F	TGTGCAGCCAAGAATCTGTA	-	SNPstream panel IV	-
rs224230b_R	CTCAGTGGTCAGCTGGAAAT	-	SNPstream panel IV	-
rs224230_GA_U1	-	ACGCACGTCCACGGTGATTTCATGACAATAATTTGTGATTTTACT	SNPstream panel IV	-
rs8054015b_F	AAGATTCTGCCACTGCACTC	-	SNPstream panel IV	-
rs8054015b_R	CTGCTTTATAACTTGCCTTTTCA	-	SNPstream panel IV	-
rs8054015_GA_U2	-	GGATGGCGTTCCGTCCTATTATATGTTTATACAGTGGAAGCCAAC	SNPstream panel IV	-
rs250470b_F	GTACTCCTGCCTGGGTGAC	-	SNPstream panel IV	-
rs250470b_R	CTAAACTTGCCTGGTGGAGA	-	SNPstream panel IV	-
rs250470_GA_U3	-	CGTGCCGCTCGTGATAGAATATTAATTAATTAATTTCCTAGTCC	SNPstream panel IV	-
rs224208b_F	ATACCTCCCTGTCCTCTGCT	-	SNPstream panel IV	-
rs224208b_R	CATATGCCTTCCTGATCTGC	-	SNPstream panel IV	-
rs224208_GA_U4	-	AGCGATCTGCGAGACCGTATTGGAGCAGGTGTACTACTTCCTGGA	SNPstream panel IV	-
rs224243b_F	TCCATGTTCCAGGACAGACT	-	SNPstream panel IV	-
rs224243b_R	CACGGAAATTCAGAAACACC	-	SNPstream panel IV	-
rs224243_GA_U5	-	GCGGTAGGTTCCCGACATATGTCATGGGTGACAGCCTTCCTTCAG	SNPstream panel IV	-
rs224215b_F	AGCCTCCTTTGCAGTTAGGT	-	SNPstream panel IV	-
rs224215b_R	CCTCAAGCTTCTGCTCTCAG	-	SNPstream panel IV	-
rs224215_GA_U6	-	GGCTATGATTCGCAATGCTTTTCTAGAGCTTTCCCRAGGGATACT	SNPstream panel IV	-
rs224241b_F	GCTCCAGAGCAGAAACTGAA	-	SNPstream panel IV	-
rs224241b_R	TGCCCTCTACAGGACAGAAA	-	SNPstream panel IV	-
rs224241_GA_U7	-	AGGGTCTCTACGCTGACGATGATAAACTGAAATGACTGACAGGCC	SNPstream panel IV	-
rs6501170b_F	TTAACCGATGCCACTGAACT	-	SNPstream panel IV	-
rs6501170b_R	CCTCCCTGGAGTATCCATTT	-	SNPstream panel IV	-
rs6501170_GA_U8	-	GTGATTCTGTACGTGTCGCCGTATCCCTTCCGGACCACTCTCATT	SNPstream panel IV	-
rs190081b_F	TGTACCCAAATATGCACGTCT	-	SNPstream panel IV	-
rs190081b_R	TTAGCAGTGTGCCTTTGACA	-	SNPstream panel IV	-

rs190081_GA_U9	-	GACCTGGGTGTCGATACCTACAGTTGTTAAGAGTGGCAATGGCCT	SNPstream panel IV	-
rs224217b_F	GAATCTCAACCCCATGATGA	-	SNPstream panel IV	-
rs224217b_R	TGCACAGCTTCACAAATGTC	-	SNPstream panel IV	-
rs224217_GA_U10	-	AGATAGAGTCGATGCCAGCTGTCTTCCCCAGATTTTCTACCTGGT	SNPstream panel IV	-
rs442387b_F	GGATACAACGCACAAACACA	-	SNPstream panel IV	-
rs442387b_R	CTCTGGTGACTCCCATAGGTT	-	SNPstream panel IV	-
rs442387_GA_U11	-	AGAGCGAGTGACGCATACTAGCAGTGTAGAAGTTAGCAAGCTGGA	SNPstream panel IV	-
rs1231124b_F	ACTTTTCAGGGACAGGCACT	-	SNPstream panel IV	-
rs1231124b_R	CCTCTGAATCCAGGGAAGAC	-	SNPstream panel IV	-
rs1231124_GA_U12	-	CGACTGTAGGTGCGTAACTCCTGGAAATGAACTACATTCTCCACA	SNPstream panel IV	-
rs250470_F	ACTTGCCTGGTGGAGAACAG	-	Fluorescence Polarization	-
rs250470c_R	ATCCCTTCCTGATGCCTTCT	-	Fluorescence Polarization	-
rs2504070_CT_Sense	-	ACACATGAAATAAAACAGTTGGG	Fluorescence Polarization	-
rs2504070_GA_AS	-	TTAATTAATTAATTAATTTTCCTAGTCC	Fluorescence Polarization	-
R26Q_F	CACTGAATTTGGAGGAGAAAATG	-	Fluorescence Polarization	Below 5% minor allele frequency
R26Q_R	ACCACATGTAGACCGCATCA	-	Fluorescence Polarization	Below 5% minor allele frequency
R26Q_GA_Sense	-	GTTCTAAGTAGAATTGAAGCTGGAC	Fluorescence Polarization	Below 5% minor allele frequency
R26Q_CT_AS	-	TTCATCCATTACACTTTTAAGTGAT	Fluorescence Polarization	Below 5% minor allele frequency
rs34236132_V534I_F	TACAAGTGCCCTGAGTGTGG	-	Fluorescence Polarization	Below 5% minor allele frequency
rs34236132_V534I_R	GGGCTCCATGGTTTGTAAGA	-	Fluorescence Polarization	Below 5% minor allele frequency
rs34236132_V534I_GA_Sense	-	TTCTCTCGGAGTTCACACCTC	Fluorescence Polarization	Below 5% minor allele frequency
rs34236132_ V534I_CT_AS	-	CTCATGAGTTCTTTCGTGAATGA	Fluorescence Polarization	Below 5% minor allele frequency
rs220379_C310S_F	TTCTGGGCTGCTGACCTAAC	-	Fluorescence Polarization	-
rs220379_ C310S_R	TCATGAGGTCTTCCCAGCTC	-	Fluorescence Polarization	-
rs220379_ C310S_GC_Sense	-	TGGAAGGTGTTCCGTCTGTAT	Fluorescence Polarization	-
rs220379_ C310S_CG_AS	-	TGAGGGTGGATGTTCTCAGAG	Fluorescence Polarization	-
N248H_F	TAAGGCGCGACAGACACTC	-	Fluorescence Polarization	Below 5% minor allele frequency
N248H_R	CTGCCAATCAGACCAACAGA	-	Fluorescence Polarization	Below 5% minor allele frequency

N248H_AC_Sense	-	CGGGAGCACGCGAGCC	Fluorescence Polarization	Below 5% minor allele frequency
N248H_TG_AS	-	GCTAATCCGCCCTCCAAT	Fluorescence Polarization	Below 5% minor allele frequency
rs224230c_F	CACAACATCGTCTCCTGCAT	-	Fluorescence Polarization	-
rs224230c_R	GAGCTGGGTGTTCAGCTAGG	-	Fluorescence Polarization	-
rs224230_GA_Sense	-	CATGACAATAATTTGTGATTTTACT	Fluorescence Polarization	-
rs224230_CT_AS	-	TGACATCCATGGGTAGTCATTAA	Fluorescence Polarization	-
rs3743930_E148Q_F	AAAACGGCACAGATGATTCC	-	Fluorescence Polarization	Below 5% minor allele frequency
rs3743930_E148Q_R	TCTCTCTGCGTTTGCTCAGG	-	Fluorescence Polarization	Below 5% minor allele frequency
rs3743930_E148Q_GC_Sense	-	TGCGGTGCAGCCAGCCC	Fluorescence Polarization	Below 5% minor allele frequency
rs3743930_ E148Q_CG_AS	-	CAGCCCCCTCCCGGCCT	Fluorescence Polarization	Below 5% minor allele frequency
rs182674_F	TCCGATTGAAAAAGGCAATC	-	Fluorescence Polarization	-
rs182674_R	AAAGAAGATGGCCAGGGTTT	-	Fluorescence Polarization	-
rs182674_GA_Sense	-	AGGCGCTGTCCCCAGC	Fluorescence Polarization	-
rs182674_CT_AS	-	ATGCGATGTAGGAAGAAGCA	Fluorescence Polarization	-
rs224217c_F	CAAATTTCAGGTAGTTCTTGGACAC	-	Fluorescence Polarization	-
rs224217c_R	GATGATGCATTGCTGTGAGG	-	Fluorescence Polarization	-
rs224217_CT_Sense	-	GAATCCAAGGGGTTTATGGA	Fluorescence Polarization	-
rs224217_GA_AS	-	CCCCAGATTTTCTACCTGGT	Fluorescence Polarization	-
rs224213_F	TCTGTGTAAGCAACTTGGGTTTG	-	Fluorescence Polarization	-
rs224213_R	ACAGGTACCGTCAACTGGGTCT	-	Fluorescence Polarization	-
rs224213_CT_Sense	-	GGCTGCGAGTCCCCG	Fluorescence Polarization	-
rs224213_GA_AS	-	TTCCTGGGCGTGGCA	Fluorescence Polarization	-
rs224208c_F	AGCCCACCTCTTATCCACCT	-	Fluorescence Polarization	-
rs224208c_R	CACCTGCAGAAGTTCCCATT	-	Fluorescence Polarization	-
rs224208_GA_Sense	-	AGCAGGTGTACTACTTCCTGGA	Fluorescence Polarization	-
rs224208_CT_AS	-	CACAAAGAATGCTCYTGCTG	Fluorescence Polarization	-

rs224205b_F	TGCTCCACTTCCACTGACAC	-	Fluorescence Polarization	Marker did not work
rs224205b_R	TCTCCCCCATATGCTTTCTG	-	Fluorescence Polarization	Marker did not work
rs224205_GA_Sense	-	AAGTCCAGGAGCCCAGAA	Fluorescence Polarization	Marker did not work
rs224205_CT_AS	-	GGCTGACTCCTGGCCTCTA	Fluorescence Polarization	Marker did not work
rs8054015_GA_Sense	-	ATATGTTTATACAGTGGAAGCCAAC	Fluorescence Polarization	-
rs8054015_CT_AS	-	TTAAAGTATCTCTTTTCAATGGTTC	Fluorescence Polarization	-
rs401298b_F	CGGGTCCAAATCTTACCTCA	-	Fluorescence Polarization	-
rs401298b_R	AGACCCAATCCTCACCCTCT	-	Fluorescence Polarization	-
rs401298_GA_Sense	-	TGTCTTACAAACCAAATTGGAAG	Fluorescence Polarization	-
rs401298_CT_AS	-	GATGTTATATTCTTGCCTTTTGTAA	Fluorescence Polarization	-
