# Supplementary material for: Genetic Variation in the Familial Mediterranean Fever Gene (MEFV) and Risk for Crohn's Disease and Ulcerative Colitis
Source: PLoS One. 2009 Sep 28;4(9):e7154. doi: 10.1371/journal.pone.0007154 (PMC2745755; doi:10.1371/journal.pone.0007154)
Supplement: Table S5 — List of oligos used to amplify fragments for the sequencing experiments. (0.14 MB DOC) [file pone.0007154.s008.doc]

**Table S5:** *List of oligos used to amplify fragments for the sequencing experiments*

| **Oligo ID** | **Sequence** |
| --- | --- |
| MEFVprom1F | GGGTTTGGGCTCAGAAAGAT |
| MEFVprom1R | CCAAACGTCAAACCACTTCA |
| MEFVProm2_F | TCAGTGGTCAGCTGGAAATG |
| MEFVProm2_R | GGCAGGAGAATCACTTGAACA |
| MEFVprom3F | CCCAAGGTGGAGTACAATGG |
| MEFVprom3R | CAAGTCTGCAAGGGAAGGTC |
| MEFVexon1F | GGCAGGAAGGAAGATTGGAG |
| MEFVexon1R | AGCTGCTCTGAGCTCCTGGT |
| MEFVexon2F | GGGGATTCTCTCTCCTCTGC |
| MEFVexon2R | GATTACAGGCATGAGCTATCG |
| MEFVexon3F | GGGGAGGACAAGCTAGGAAG |
| MEFVexon3R | TAATGCACCAACAACCCAGA |
| MEFVexon4F | CCTCAGCCTTGCTACCAGAA |
| MEFVexon4R | TCTGTCCCCTGAGAGGAGGT |
| MEFVexon5F | CTGGGGGTTCCTGGACAT |
| MEFVexon5R | GGTCACCAAGACCAAGTCCT |
| MEFVexon6F | GCCTGAATTCCCGTGGTTAG |
| MEFVexon6R | GAACATCTCCCTCCCAGGTC |
| MEFVexon7-8F | TGTAGTTCATTTCCAGCTCACG |
| MEFVexon7-8R | TTCTAAATAGGGCCCCTCAA |
| MEFVexon9-10aF | CTAAGCAGGGGGTTCCTTGT |
| MEFVexon9-10aR | AATAAAGGAGCCTCCCAAGC |
| MEFVexon9-10bF | AGAATGGCTACTGGGTGGTG |
| MEFVexon9-10bR | AATCCAGTCTGCTTGCGTTT |
| MEFVexon9-10cF | AACAGAAGATTTGGCCCTCA |
| MEFVexon9-10cR | GTGGCTCATGCCTGTAATCC |
| MEFVexon9-10dF | GTAGAGACGGGGGTTTCACC |
| MEFVexon9-10dR | CATGAGGCCCAGTCAATTCT |
| AK096958prom1_F | CTGAGATGAGGGGACACTGG |
| AK096958prom1_R | GTGGGTTGGTCCAAGTTGAG |
| AK096958prom2F | TTGCTGAGTGTGGTGGTGAT |
| AK096958prom2R | TGGTGTGGGGATCTATTCAGA |
| AK096958prom3F | CTCCTGAGCAGCTTGGAAAC |
| AK096958prom3R | GCCTGTAATCCCACCACTTT |
| AK096958exon1aF | GTCAGGCTGGTCTCGAACTC |
| AK096958exon1aR | ACACTGCGGACAGTTCCTTT |
| AK096958exon1bF | GGAGGAGACGTGTGGAAAGA |
| AK096958exon1bR | TCTCAGCCTCCTTGGAACAG |
| AK096958exon1cF | CTGTGCTGACCCCAAAACC |
| AK096958exon1cR | GTCAGGAGAGCGAGACCATC |
| AK096958exon1dF | CCTTCCGAGTAGCTGGGATT |
| AK096958exon1dR | AGTGCCCTGGTGACAGACAT |
| AK096958exon2F | ATGACTGACAGGCCATTTGC |
| AK096958exon2R | GAACCCCCATTTTACAGTTGA |
| ZNF263prom1F | TTCAGGAACTTGCTGTGCAT |
| ZNF263prom1R | CTGCCTCAGCCTCCTGAGTA |
| ZNF263prom2F | TGGTGAAACCCCGTCTCTAC |
| ZNF263prom2R | TTTAAATCTGCCCGAGATGTG |
| ZNF263prom3F | CCCCCAACTATCACAGTCCA |
| ZNF263prom3R | TGAAATTCTGGGAGTGACTGG |
| ZNF263exon1aF | GACACTGACGCACTGGAGAG |
| ZNF263exon1aR | CAGTCCTCCTCCAGCTTCAC |
| ZNF263exon1bF | TCCAACCTTACATGGGTTCA |
| ZNF263exon1bR | CAAGTCCTCTTCCCCACGTA |
| ZNF263exon2F | GATCGCCTGAGGTTCTCTGT |
| ZNF263exon2R | CACCAACTGCAGGTCTTTCA |
| ZNF263exon3-4F | AGCTGATTAGGCCTCTGTGC |
| ZNF263exon3-4R | AGGCTTCTTGGCAAAACTCA |
| ZNF263exon5F | TCCACTGGGAACAGTTTGTG |
| ZNF263exon5R | TGCCTCACTCTTAACTGCAAGA |
| ZNF263exon6aF | TTCTGGGCTGCTGACCTAAC |
| ZNF263exon6aR | CCCACACTCGGGACACTTAT |
| ZNF263exon6bF | GCACCAGAGAACGCACACT |
| ZNF263exon6bR | CTCTGCACCTCCCTCACAAT |
| ZNF263exon6cF | TGAGGTGGCATATTCAGAGG |
| ZNF263exon6cR | GATCAACACTCGGGGGTCTA |
| TIGD7prom1F | CAGTAGCGCCATCAGCATTA |
| TIGD7prom1R | CAGGCCACTATGACAGTTGTGT |
| TIGD7prom2F | AAAAAGACGACGGCACAGC |
| TIGD7prom2R | GCCATCTCGGCTCACTACA |
| TIGD7prom3F | AAAAATTAGCCGGGTGTGGT |
| TIGD7prom3R | TTCTCAGAGATGAGCCCAAAA |
| TIGD7exon1aF | CACTGAAGAGAAATCAAAATCTCAA |
| TIGD7exon1aR | CAAGATGTGCCCACAAGACA |
| TIGD7exon1bF | CTTTTTGGAAGGGGAGAAGC |
| TIGD7exon1bR | CTTACTGATTCCAAATTCATCCA |
| TIGD7exon1cF | TCTCCTGTTGGTGGTTCAGA |
| TIGD7exon1cR | TTGGGCAGTTTTGATTTTCC |
| TIGD7exon1dF | CAGTGGGGATGAAACAGACC |
| TIGD7exon1dR | TCCCCATGTTCTAAGCCTTG |
| TIGD7exon1eF | GGCAAAAAGTTGGGAAGAAG |
| TIGD7exon1eR | GCAAAGGCATTCCACCTTAG |
| ZNF75Aprom1F | TGCTAGCAGAACTTCACTTATTCAA |
| ZNF75Aprom1R | CGCCTGTAGTCCCAGCTACT |
| ZNF75Aprom2F | TTTTGCGACGGAGTCTCG |
| ZNF75Aprom2R | TGTAGCCAATATTGCAGAGTTGA |
| ZNF75Aprom3F | CCCAACCCCAAATCAACTT |
| ZNF75Aprom3R | TCGAGGAAGAGGCATTTTGT |
| ZNF75Aexon1F | TGCGTCACGAGACCTAGAAA |
| ZNF75Aexon1R | CACCGCCTACGGAAAATG |
| ZNF75Aexon2F | CAATGTGGGCTGTACACCTG |
| ZNF75Aexon2R | TTGCTATCAAGGCTGGTTTT |
| ZNF75Aexon3F | CTAGTTCTAGGGGCTGGTGGA |
| ZNF75Aexon3R | AGCCTGGGAGGTACCCAGTT |
| ZNF75Aexon4F | CTTTATCGCCTGGCCAATTC |
| ZNF75Aexon4R | CCTACACCCTCCCCTATACCA |
| ZNF75Aexon5F | TCCATTTAACCATTTGGAGTAACA |
| ZNF75Aexon5R | GCCCTTGCAAAAGTTCACAT |
| ZNF75Aexon6aF | CCTGTGATTTGCTTGTTCTGG |
| ZNF75Aexon6aR | CCAAGCTCCATTTGGTGAAT |
| ZNF75Aexon6bF | AGACACCAGAAACTCCACCTG |
| ZNF75Aexon6bR | TGGGCAAGTACTAATTTTTATAGCTG |
| ZNF75Aalt1aF | TCATAAGTCGCAAGTGATGAGG |
| ZNF75Aalt1aR | AAGCCAGACTAGGCCCAAC |
| ZNF75Aalt1bF | GGACAGTTAAGGTGGGACTTGA |
| ZNF75Aalt1bR | CTTGGCCTCTCAAAGTGCTG |
